# Machine learning based classification of mitochondrial morphologies from fluorescence microscopy images of Toxoplasma *gondii* cysts

**DOI:** 10.1371/journal.pone.0280746

**Published:** 2023-02-02

**Authors:** Brooke C. Place, Cortni A. Troublefield, Robert D. Murphy, Anthony P. Sinai, Abhijit R. Patwardhan

**Affiliations:** 1 F. Joseph Halcomb III, M.D. Department of Biomedical Engineering, University of Kentucky, Lexington, Kentucky, United States of America; 2 Department of Microbiology, Immunology & Molecular Genetics, University of Kentucky, Lexington, Kentucky, United States of America; Foshan University, CHINA

## Abstract

The mitochondrion is intimately linked to energy and overall metabolism and therefore the morphology of mitochondrion can be very informative for inferring the metabolic state of cells. In this study we report an approach for automatic classification of mitochondrial morphologies using supervised machine learning to efficiently classify them from a large number of cells at a time. Fluorescence microscopy images of the chronic encysted form of parasite *Toxoplasma gondii* were used for this development. Manually classifying these morphologies from the hundreds of parasites within typical tissue cysts is tedious and error prone. In addition, because of inherent biological heterogeneity in morphologies, there can be variability and lack of reproducibility in manual classification. We used image segmentation to detect mitochondrial shapes and used features extracted from them in a multivariate logistic regression model to classify the detected shapes into five morphological classes: Blobs, Tadpoles, Lasso/Donuts, Arcs, and Other. The detected shapes from a subset of images were first used to obtain consensus classification among expert users to obtain a labeled set. The model was trained using the labeled set from five cysts and its performance was tested on the mitochondrial morphologies from ten other cysts that were not used in training. Results showed that the model had an average overall accuracy of 87%. There was high degree of confidence in the classification of Blobs and Arcs (average F scores 0.91 and 0.73) which constituted the majority of morphologies (85%). Although the current development used microscopy images from tissue cysts of *Toxoplasma gondii*, the approach is adaptable with minor adjustments and can be used to automatically classify morphologies of organelles from a variety of cells.

## Introduction

There is an increasing interest in assessment of metabolic state at the cellular level in a variety of fields. In many of these studies there is a need for classification of the morphology, i.e. form, of the mitochondrion, which is the subcellular organelle that plays a key role in cellular metabolism. In the present study, we developed an automated classification approach that could classify the morphologies of the mitochondrion within the poorly understood chronic form of the parasite *Toxoplasma gondii (T*. *gondii)*, termed a bradyzoite. Bradyzoites, numbering in the hundreds are resident within tissue cysts, typically in the central nervous system and muscle. The motivation for this development was that although these bradyzoites were long considered to be non-replicative and metabolically quiescent [1], our prior work demonstrated that in vivo they exhibit a surprising level of metabolic activity including the ability to replicate [2]. This form is additionally resistant to current drug treatments, making insights into their physiology particularly relevant [3]. Inherent to parasite’s viability and the potential for growth is a requirement for energy which is derived from the activity of a single mitochondrion [4] the morphology of which is reflective of the parasite’s physiological state [5, 6].

*T*. *gondii* chronically infects a third of individuals globally [7]. In most healthy individuals, the initial infection is asymptomatic and readily controlled by the immune system. However, the immune system does not get rid of the parasite; instead, the parasites enter a second life-cycle stage in tissue cyst that house slow growing bradyzoites. These tissue cysts retain the potential to reactivate in the immunocompromised, especially in the case of HIV-AIDS, which can result in toxoplasmic encephalitis that is lethal if not treated [7].

Image processing and machine learning methods have been successfully used by others in mammalian cells to classify normal mitochondria [8], the effects of various stressors including drug treatment on mitochondrial morphology [9, 10] and mitochondrial dynamics and plasticity [11, 12]. In contrast to the mammalian cells which contain networks of mitochondria, Toxoplasma gondii cells contain a single mitochondrion [4] which in the actively growing tachyzoite form exhibits considerable plasticity [5, 6]. However, the status and dynamics of mitochondrial morphology have not been investigated in bradyzoites within tissue cysts. This is partially due to the high demand of time and trained specialists that would be required to perform this manual process of classifying potentially thousands of mitochondrial objects from fluorescent microscopy images of cysts containing bradyzoites. In addition, biological heterogeneity introduces ambiguity which makes manual classification of some objects difficult leading to variation in classification by expert users and potential lack of reproducibility in classification. In this study, we used image segmentation to detect fluorescently labelled mitochondrial objects and nuclei within microscopy images of tissue cysts. For a subset of these images, trained specialists manually classified (labeled) each identified mitochondrial object’s morphology within the five predefined classes. Features related to intensity, size, shape/ texture, and spatial relation to nearby nuclei were extracted from these objects and used in a supervised machine learning approach to classify their morphologies. A small subset of these results related to congruence among operators in manual classification of objects were previously reported in a conference proceedings paper [13].

## Methods

**Fig 1:**
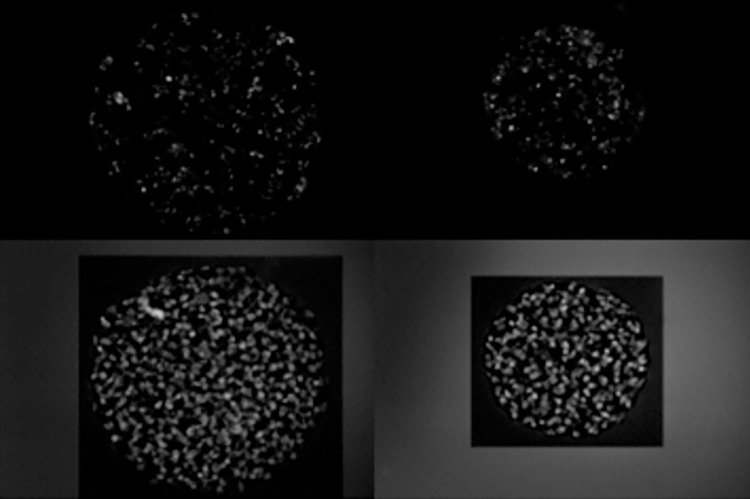
Examples of images showing actively respiring mitochondria illuminated via MitoTracker (top), and nuclear objects.

The cysts were harvested as described previously from brains of mice infected with T *gondii* [2]. This study was carried out in strict accordance with the recommendations in the Guide for the Care and Use of Laboratory Animals of the National Institutes of Health. All experimental procedures involving use of animals were approved by the Institutional Animal Care and Use Committee (IACUC) at the University of Kentucky (protocol #2018–3083, date of approval 1/24/2019). All infections by i.p. injection were conducted on animals mildly anesthetized with 30% isoflourane using a drop jar method. Animal work in this study was restricted to the infection of mice with Toxoplasma gondii with the purpose of generating tissue cysts for analysis. As the development of tissue cysts is dependent on the symptomatic acute phase of infection, animals were not medicated with either anti-parasitic or anti-inflammatory agents. Mice were euthanized by CO_2_ asphyxiation followed by cervical dislocation, a method that was approved by the institution’s IACUC. Fluorescence microscopy images were acquired following accumulation of the mitochondrion targeting reagent MitoTracker (Invitrogen). Nuclei from the bradyzoites were stained with DNA dye DAPI (Invitrogen). An optical section of the tissue cyst illuminated the mitochondria and the nuclei as shown in Fig 1. The visualized mitochondrion and nucleus provide a spatial relationship of individual bradyzoites within labeled cysts which was found to be helpful in classifying the mitochondrial morphology, with the nucleus providing a morphological point of reference within the parasite.

The developed program performs image processing for object detection, extracts features of the detected objects and collects user input on the object’s morphological classification. The classification information collected from the user along with the extracted features for each object was then used as the gold standard to train a logistic regression algorithm. The time-consuming process that a trained user would perform of manually identifying and classifying the individual objects and their shapes from within fluorescence-based images of tissue cyst was automated by using the trained machine learning algorithm to predict the classification of objects detected in new images. The program with a customizable and user-friendly graphical user interface (GUI) was developed in MATLAB using its App Designer, Image Processing, Computer Vision, and Optimization toolboxes.

### Image processing

The 8-bit grayscale images underwent linear intensity scaling, adjusting the pixel intensity range to that of the full range of resolution, 0 to 255. Image contrast was enhanced using a top hat and bottom hat filter [14] as described in Eq 1. The addition of the top hat filtered image to the original image emphasized the foreground making the bright spots brighter, while the subtraction of a scaled bottom hat filter enhanced the perimeter of the bright objects. In implementing the erosion and dilation processes of the filters a disc shaped structuring element was used with a radius of 10 pixels for the top hat filter and a radius of 3 pixels for the bottom hat filter.


[J]=[Tophat(I)]+[I]−10*[Bottomhat(I)] (1)

To automatically detect the objects of interest, which were the active mitochondrion (“Mito”) or nuclei (“DAPI”) the Otsu method was used to determine a starting threshold. This method minimizes interclass variance while maximizing between-class variance of the intensity distributions of the foreground and background pixel values [15]. The starting threshold was used to create binary images.

Initial evaluation of this starting threshold showed that a more conservative value was better to accurately capture the objects of interest within the Mito images, especially their shape which was the key attribute being investigated. Therefore, the default, i.e., starting threshold for the Mito image analysis was set to be equal to 40% of the Otsu threshold. Implementing this threshold separated background pixels from pixels representing the target objects and was able to segment the target objects sufficiently from each other as determined by expert users. The nuclear objects within the DAPI images appeared more defined, therefore the starting threshold for these images was defaulted to 80% of the Otsu threshold, excluding a greater number of lower value pixels and preventing under-segmentation. To better segment the nuclear objects within the DAPI images, especially those within proximity of each other, a watershed segmentation [16] was applied. In order to prevent over segmentation, which can occur with the watershed method, a height transform [17] was applied before watershed segmentation to suppress peaks of one standard deviation of the image pixel values or less. Since the threshold selection was important in appropriately segmenting the objects of interest, a slider was added to the GUI to allow for expert users to adjust the starting threshold as needed and observe the resulting changes to the binary image immediately.

To locate each individual object of interest within the Mito binary image the Moore-Neighbor tracing algorithm with Jacob’s stopping criterion was used [14]. The resulting boundaries were superimposed onto the original image to enable extraction of each object’s pixel intensities and geometric information. A total of 22 features were extracted for each object. Features related to pixel intensities were maximum, mean, minimum, and mean squared error between the original and enhanced object. Features related to the object’s size were area, perimeter, major axis, minor axis, and extent. Those related to shape and texture were circularity, eccentricity, aspect ratio, the number of dominant peaks found in the histogram of gradients, intensity variance, intensity standard deviation, hole count, size of all holes, number of segments found in the object after using watershed transform, and ratio of intensity at extrema. The last three features, distance to closest nuclei centroid, distance to closest nuclei extrema, and distance to closest mitochondrial object centroid, were related to an object’s location relative to the location of nuclei within the corresponding DAPI image.

The objects identified in the Mito image were then filtered to exclude objects that were smaller than a specified size or lower than specified pixel intensity values as they would typically be ignored in manual evaluation. Extracted features of minimum axis length and maximum intensity of each object were compared against user selected ‘low size’ and ‘low intensity’ for exclusion based on these criteria. The ‘low size’ exclusion criterion defaulted to half a micron (seven pixels) to exclude objects with a minor axis, or width, less than this value in microns. The ‘low intensity’ exclusion criterion defaulted to the same threshold used for binarization, and therefore did not exclude any objects unless increased by the user to exclude objects that were dimmer and may have occurred due to over segmentation. Once these exclusion criteria were updated the user could observe their effect as the objects excluded would not have a colored bounding box around them within the original or binary images shown in the GUI.

**Fig 2:**
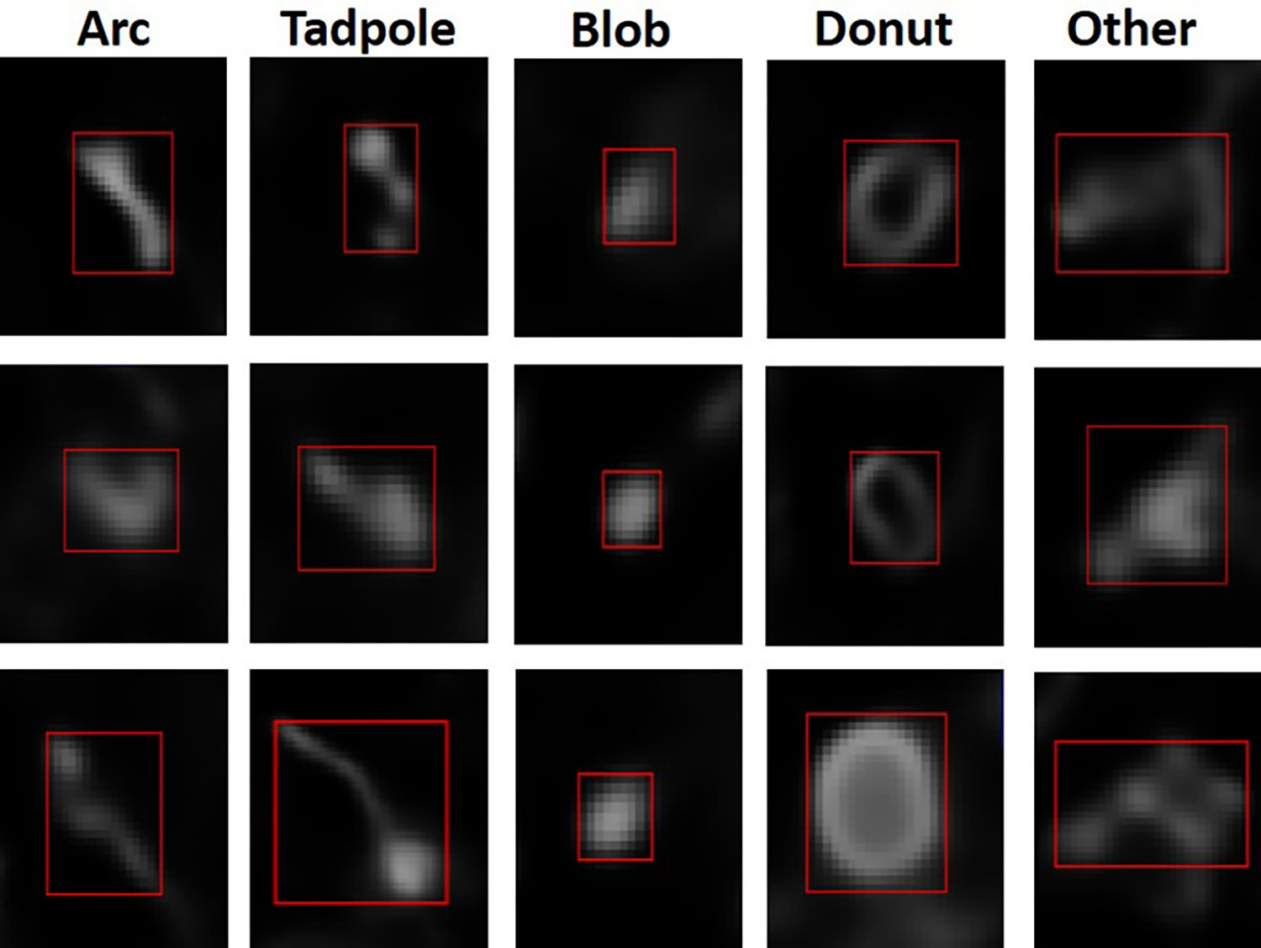
Representative images for different classes of observed mitochondrial morphologies in encysted bradyzoites.

We used the mitochondrial profiles observed in tachyzoites, which is the fast growing form of *T gondii*, as the main morphologies to classify the objects identified in the Mito images because nothing is currently known about these morphologies in bradyzoites. These included Lasso (ring), Arcs (linear and curvilinear), Tadpoles (sperm-like) and Blobs (collapsed) forms [5, 6]. Sample images of each class are depicted in Fig 2. After initial analysis several objects appeared with a similar shape to the Lasso class but were markedly smaller in size. These were referred to as “donut” shape, and a Donut class was added. Although the Lasso class, associated with actively replicating intracellular tachyzoites [4–6] were not observed in the tissue cysts imaged during the development of this application, its detection remains a possibility with larger sample sizes.

Three expert users were involved in providing user feedback and manually classifying objects by providing the labels to be used during the machine learning phase. Initially, as a group, the users developed the following qualitative and quantitative descriptors to best define the mitochondrial morphological classes, these descriptors helped guide selection of features to be used in the machine learning approach.

The Lasso/donut class appeared as ring or loop shaped with a visible hole in the middle that is considered to reflect the normal morphology of a functioning mitochondrion in actively growing tachyzoites [4–6]. Notably, while lasso forms are large, often tracing out the outline of the parasite while enclosing the nucleus [4–6], donuts which are significantly smaller and do not encircle the nucleus, may represent a feature of bradyzoites. The Tadpole class (also described as sperm like [6]), has a bulbous bright end with a tail or linear string like protrusion that is often thinner, fainter, and equal to or greater than the diameter of the head. The Arc class can be dumbbell shaped with fairly uniform arms and a thin connection; it is often connected in the shape of an arc but may also include more linear shapes. The Blob class were punctate, circular in shape, ranging in brightness, and typically smaller (between 0.5 microns and 1 micron in diameter). An ‘Other’ class was added to include those not belonging to the above classes because the morphological diversity of encysted bradyzoites’ mitochondrial profiles has not been analyzed in detail before. These tended to include more complex and morphologically diverse branched forms.

### Capturing manual classification

The developed program automatically identified the objects of interest and isolated them for manual classification that were used as labels during machine learning. A GUI was developed based on feedback from the users to facilitate the process [13]. The user interface displayed the Mito image and the corresponding DAPI image. First, the default settings were reviewed and updated, if needed, by a designated lead user to best identify each mitochondrial object. These settings were used by all users who subsequently performed independent manual classification to ensure that they were classifying the same objects.

### Machine learning for classification

For the automation of the classification of mitochondrial morphologies, a supervised logistic regression algorithm was implemented. The linear regression hypothesis (Eq 2) was used with the sigmoid function (Eq 3) to determine the probability that a given object’s features were associated with a class on a scale of 0 to 1 [18]. Each class was evaluated individually to determine the probability of the object belonging to the specific class, then compared to the probability determined for each class to select the class with the maximum likelihood. This was done by creating a gold standard vector Y, to consist of 1’s for the class being evaluated and 0’s for all other classes.

$$h(x^{M})=\theta_{0}x_{0}^{M}+\theta_{1}x_{1}^{M}+\cdots \;\theta_{N}x_{N}^{M}$$ (2)

$$g(z)=\frac{1}{1+e^{-z}}$$ (3)

in the equation above, $x_{0}^{M}$ = 1 and θ_N_ parameter sets the contribution from each of the N features. x is a matrix composed of M rows, one for each object, and N+1 columns of features with the value of $x_{0}^{M}$ = 1 added to allow for the simplification of Eq 2. h(x) is a column vector with M rows, each row representing the predicted class for each M object. *θ* is a parameter row vector with the first column, *θ*_0_, being constant with a value of 1, and the following columns representing the coefficiants defining the contribution of each N feature.

Since the different features had varying numerical ranges, the features were first normalized, i.e. scaled using Eq 4, to allow for the efficient calculation of the optimum ***θ*** parameter to minimize the cost function (Eq 5). The cost function determines the error or how closely the hypothesis (i.e. predicted class from Eq 2) compares to the gold standard (Y) [18].


$$X_{N}^{M}=\frac{X_{N}^{M}-mean(X_{N})}{std(X_{N})}$$ (4)

$$J(\theta )=\frac{1}{M}\sum_{i=1}^{M}[-Y^{\left(i\right)}\;\log\left(h(X^{\left(i\right)})\right)-(1-Y^{\left(i\right)})\;\log\left(1-h(X^{\left(i\right)})\right)]+\frac{\lambda }{2M}\sum_{j=1}^{N}\theta_{j}^{2}$$ (5)

$$\left[superscript\;(i)\;represents\;the\;i^{th}\;row,or\;object\right]$$

A regularization parameter, λ, was included in the cost equation to prevent overfitting and to generalize the model for future predictions by adding a cost for the parameter vector used and limiting the algorithms overall fit to the training set. Optimal θ parameters were found using the function ‘fminunc’ in MATLAB which implements a quasi-Newton method [19]. An optimal θ parameter vector was obtained for each class, essentially creating 5 models for prediction. Then using the logistic regression (Eq 2) trained for each class a probability for each class was evaluated for every M object as described in Eq 6, which selects the class associated with the model that resulted in the greatest value as the prediction [18, 20].


$${prediction}^{M}=\max[h_{blob}(X^{M}),\;h_{tadpole}(X^{M}),\;h_{donut}(X^{M}),\;h_{arc}(X^{M}),\;h_{other}(X^{M})]$$ (6)

The prediction vector, *prediction*^*M*^, was then compared with the gold standard vector Y, to determine the overall accuracy or percent of all objects that were correctly classified as well as the sensitivity, specificity, precision, and F score per class as described in Eqs 7–10 to analyze the trained algorithm’s performance.


$$Sensitivity=\frac{True\;Positives}{True\;Positives+False\;Negatives}$$ (7)

$$Specificity=\frac{True\;Negatives}{True\;Negatives+False\;Positives}$$ (8)

$$Precision=\frac{True\;Positives}{True\;Positives+False\;Positives}$$ (9)

$$F\;score=\frac{2*Sensitivity*Precision}{Sensitivity+Precision}$$ (10)

### Validation of classification

The manual classification, machine learning based automatic classification and validation of automatic classification was performed in three steps. The first step used a set of 5 randomly chosen images to determine whether certain morphologies were readily classifiable and to evaluate the congruency between users’ classification. The results showed the agreement between 2 of the 3 users to be remarkably high (96%) and modest between all 3 users (57%) [13].

The second step involved classifying the objects within the initial 5 images with all three users as a group to obtain a consensus “gold standard” classification to be used to train the machine learning algorithm. We also obtained manual classifications for 5 new images to evaluate (blinded) the trained algorithms’ performance. To determine the effect, if any, of changing the settings during segmentation and isolation of objects, the effects of the changed settings chosen by the lead user at a separate time for the first 5 images were evaluated and the 2 images where the largest differences in object counts were observed were selected for further analyses. These 2 images were classified twice, once with the initial settings and again with the new settings to evaluate the impact the change in settings had on the class distributions classified by each user. The gold standard which was used for training the machine learning algorithm and was determined in two sittings. In the first sitting all the objects from one of the initial 5 images, from the first step were reviewed. Due to having no objects that the users unanimously agreed which were Lassos and the observation of multiple Donut shaped objects the addition of the Donut class was made at this point prior to the individual classifications. In the second sitting, only the objects where all three users did not already have an agreement on from their individual classifications (for the remaining 4 images) were reviewed to determine a consensus classification to be included in the gold standard. The performance of the trained algorithm was then evaluated against the classifications made by each individual user of the 5 new images in step 2.

In the third step, we used the trained algorithm to predict the class for objects identified in 10 new images (which were not used in training of the model) from which the users individually (not as a group) selected objects at random to see what the automatic class determination for that object was and noted whether they agreed or disagreed with the classification, hence this was a non-blinded evaluation of the machine learning’s performance.

## Results

### Manual classification: Individual and consensus

The congruency between users for the first 5 images in step 1, before introduction of the Donut class showed that 57% of the objects were classified similarly by all three users, 96% were classified the same by 2 of the 3 users, and 4% of the objects had no agreement between users as to which class they should be placed [13]. In step 2 of classifications for these images after the addition of the Donut class, 71.8% of the objects were classified similarly by all users, 98.8% were classified the same by 2 of the 3 users, and 1.2% of objects had no agreed upon classification. Step 2 showed higher congruency in Blob classification as compared to the first time the objects of these images were classified in step 1. In classifications in both steps, Blobs were the most frequent class to occur as well as the most likely to be uniformly recognized by all users. There was an increase in instances in step 2 where all three users agreed for the Arc and Other classes, but the agreement for Tadpoles remained unchanged.

**Fig 3:**
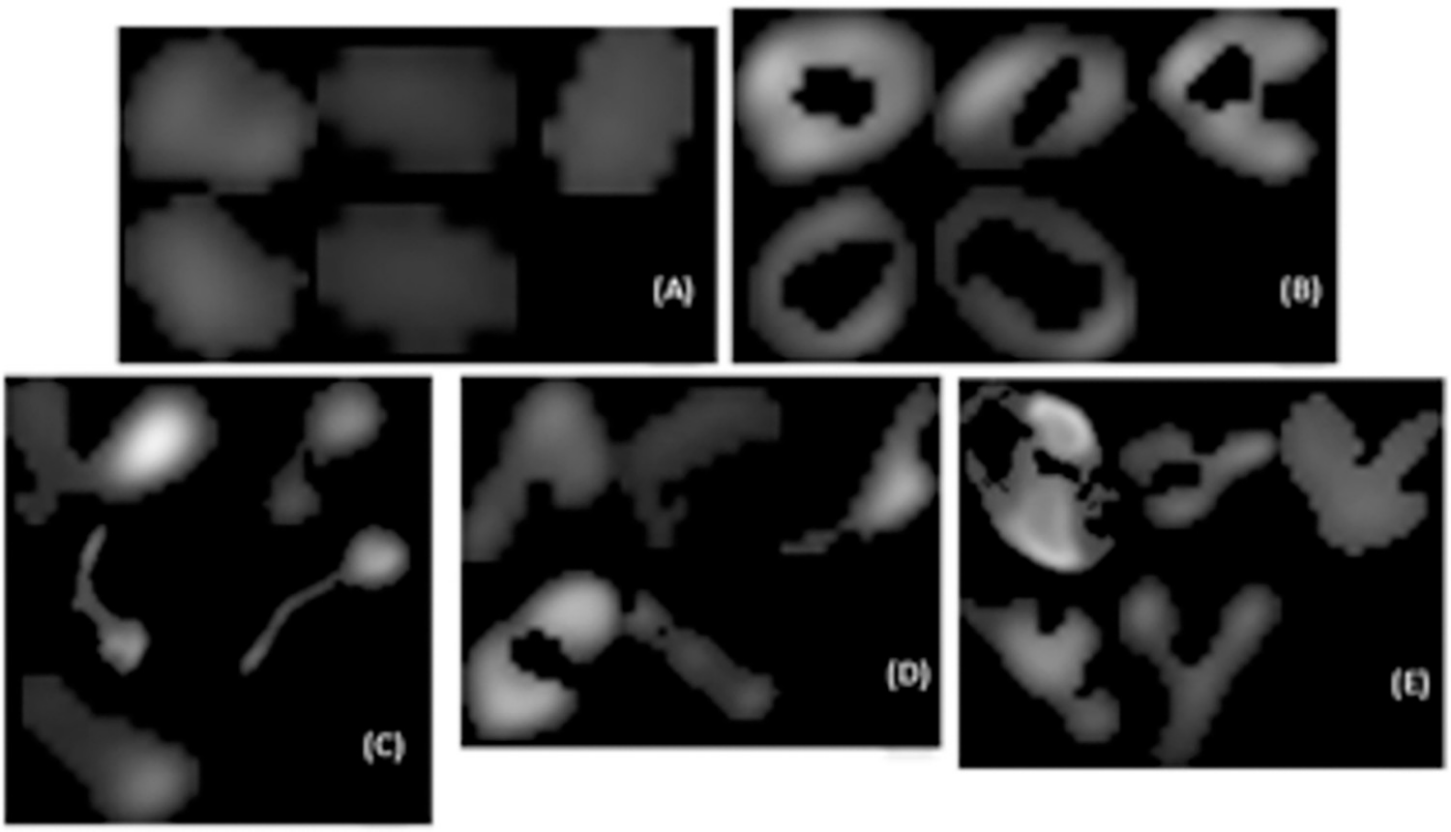
Objects classified that had agreement among all users and matched the gold standard for Blobs (A), Donuts (B), Tadpoles (C), Arcs (D), and Others (E).

For all 5 images from which a gold standard classification was determined, there were a total of 1,138 objects detected. As stated above, the majority of objects were classified as belonging to the Blob class with a count of 625, followed by 345 Arcs, 81 Tadpoles, 50 Others, and 37 Donuts. Comparing these consensus (i.e. gold standard) distributions to that of each user’s step 2 individual classification, the following matches were determined (%): 83.4 for user 1, 86.7 for user 2, and 87.1 for user 3. Objects for each class where there was 100% agreement between users and which matched the gold standard are shown in Fig 3.

**Fig 4:**
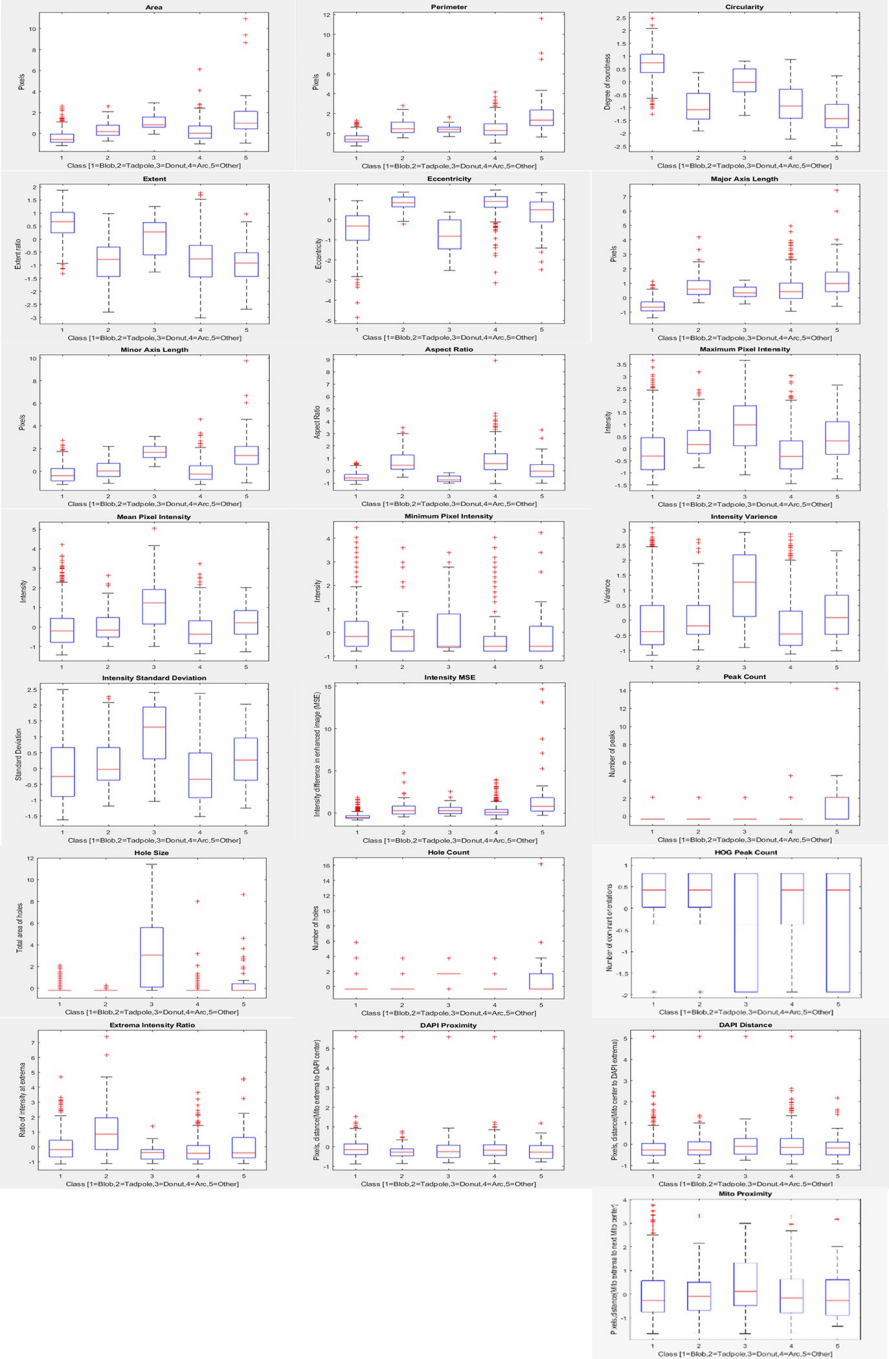
Depiction of the 22 normalized features and their distribution for each class from the 1138 gold standard objects.

To visualize the trends for each class by feature as observed in the gold standard classification for the first 5 images, Fig 4 shows a boxplot for each class for each of the 22 features. The top of the box marks the 75th percentile and the bottom the 25th percentile with a red line indicating the median, the red ‘+’ markers indicate the outliers which are greater than one and a half times the interquartile range. One observed trend was in the circularity and extent features, in which higher values were seen for the Blob class followed by the Donut class, while the eccentricity and aspect ratio features were highest for the Tadpole and Arc class. As expected, a telling feature of the Donut class was the presence of holes which correlates with the higher hole size, while a telling feature of the Tadpole class was a bright head with a narrow and/or dim tail which correlates to the higher extrema intensity ratio.

**Fig 5:**
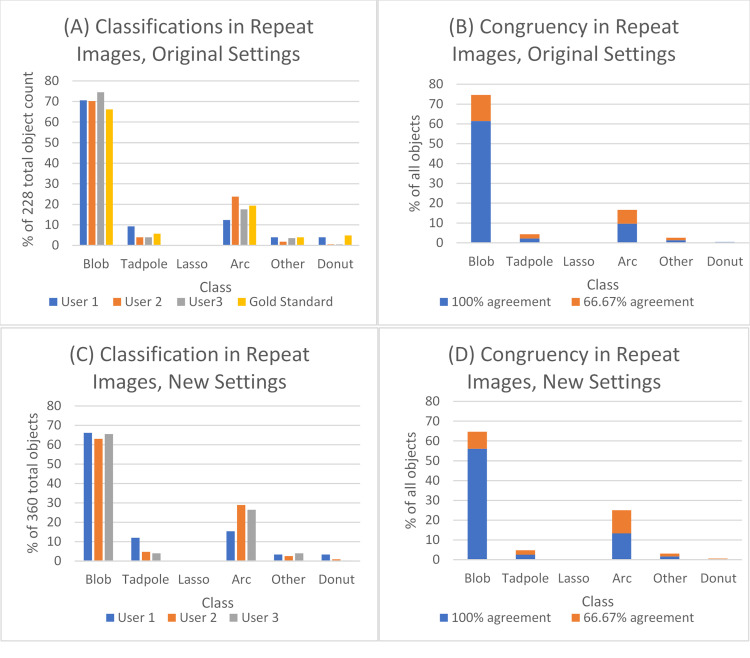
Percentage of all objects classified separated by classification and user for the 2 repeat images with original settings (A) and new settings (C). Percentage of all objects classified separated by classification and the percent agreement between users for the 2 repeat images with original settings (B) and new settings (D).

The effect of change in settings on class distribution and congruency for the 2 images that were classified twice is shown in Fig 5. The congruency was comparable; with new settings, 73.9% of the objects were classified similarly by all users, and 98.1% of the objects were classified the same by 2 of the 3 users. When the original settings were used, 75% of the objects were classified similarly by all users, and 98.7% of the objects were classified the same by 2 of the 3 users. The distribution of classes identified by each user for the images remained relatively consistent with a slight decrease in Blobs and increase in Arcs in the images with new settings.

There was a total of 927 objects that were classified from the new set of 5 images in step 2 that had not previously been analyzed. Of the 927 objects 697 (75.2%) were placed in the same class by all 3 users, i.e. they were classified similarly by all users. A total of 918 (99%) objects were classified as belonging to the same class by 2 out of the 3 users. These results consistently showed Blobs as the most frequent class to occur and a high congruency amongst users recognizing this class. The agreement for and frequency of occurrence for the Arc, Donut, and Other classes were comparable to the results from the first five images with a slight increase in congruency for Tadpoles.

### Machine learning performance

Objects from the first image from which consensus gold standard was obtained (each image consisted of one cyst), which had 334 objects, was initially used to train the machine learning algorithm. Once the optimal θ parameter arrays were determined (one for each of the five classes), referred to as Model 1, the overall training accuracy was found to be 86.8%. This was the fraction of the number of objects that were classified correctly by the algorithm regardless of class for the same 334 objects used in training. The breakdown per class, in Table 1, showed a high degree of performance for the Blob, Donut, and Arc classes. The sensitivity, or recall, is the percentage of actual positives predicted accurately (true positive rate) while specificity is the percentage of actual negatives predicted accurately (true negative rate). Precision also called positive predictive value is the number of true positives divided by the number of predicted positives. Lastly, the F score uses the calculated sensitivity and precision values to provide a single metric to compare the performance for each class [18].

**Table 1:** Training performance of model 1 using labels from the first gold standard image.

(Model 1)	Blob	Tadpole	Donut	Arc	Other
Sensitivity	0.97	0.41	0.91	0.85	0.45
Specificity	0.91	0.99	1	0.90	0.98
Precision	0.92	0.63	1	0.82	0.64
F score	0.95	0.5	0.95	0.84	0.53

**Fig 6:**
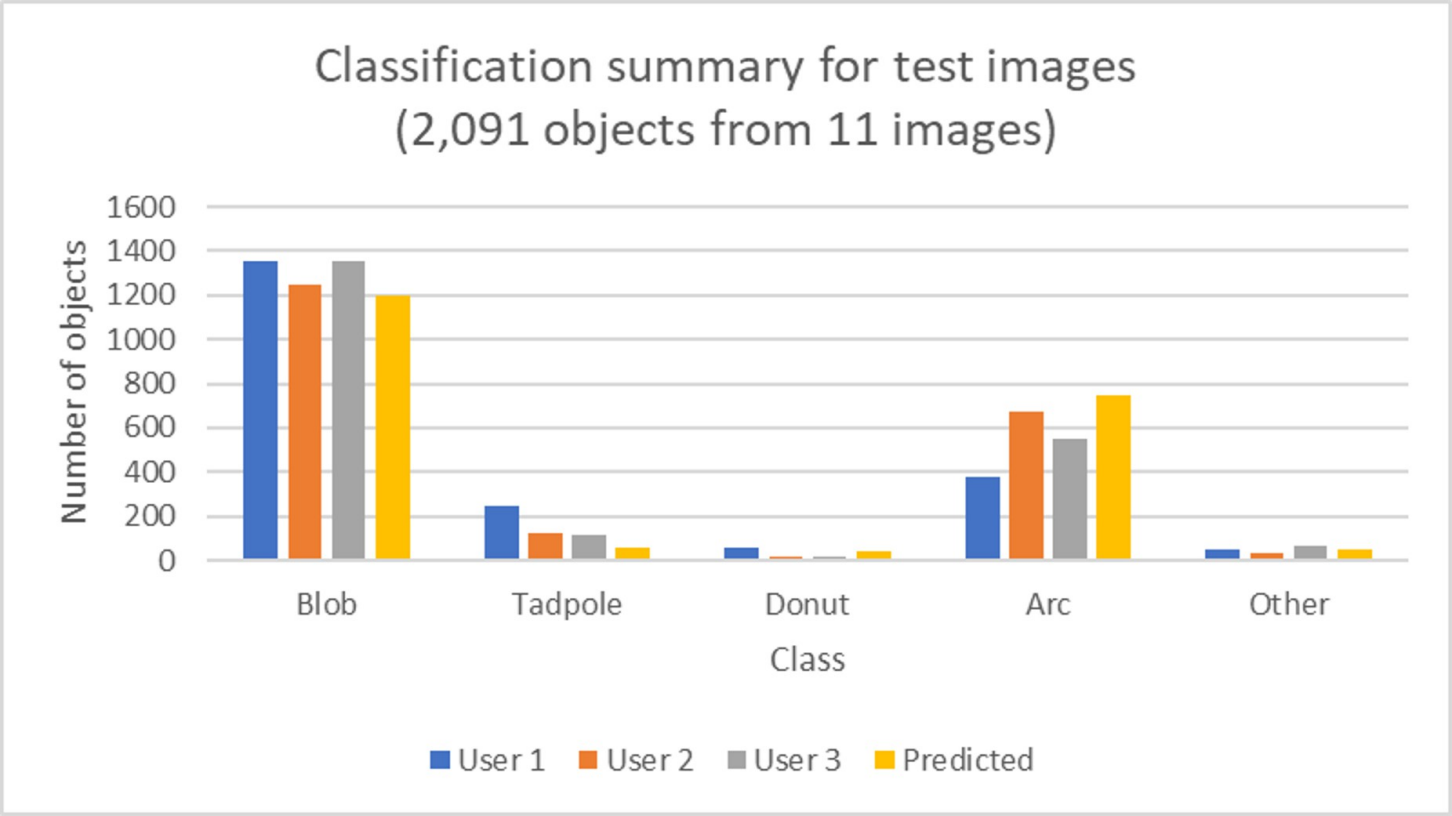
Summary of the number of objects each user classified as one of the five classes for the objects in the 11 test images along with the trained algorithm’s predicted classifications.

Testing Model 1 on the remaining 11 images (remaining 4 from initial set, 2 from the same set but with new settings, and 5 new images) that were classified individually produced an overall accuracy of 73.8%, 82.8%, and 82.6% when compared with classifications by user 1, 2, and 3 respectively. The overall accuracy was computed as the percent of objects where the predicted class matched that of the user’s classification. A summary of the objects that were identified by each user in the test set is shown in Fig 6. The performance breakdown by the user is given in Tables 2–4. The performance for Model 1 for the Blob class remained high and there was overall high specificity for all classes.

**Table 2:** Testing performance of model 1 against classifications by User 1.

(Model 1)	Blob	Tadpole	Donut	Arc	Other
Sensitivity	0.84	0.12	0.33	0.91	0.21
Specificity	0.92	0.99	0.99	0.76	0.98
Precision	0.95	0.56	0.53	0.45	0.21
F score	0.89	0.20	0.41	0.60	0.21

**Table 3:** Testing performance of model 1 against classifications by User 2.

(Model 1)	Blob	Tadpole	Donut	Arc	Other
Sensitivity	0.89	0.27	1	0.83	0.29
Specificity	0.89	0.99	0.99	0.87	0.98
Precision	0.93	0.62	0.42	0.75	0.19
F score	0.91	0.38	0.59	0.79	0.23

**Table 4:** Testing performance of model 1 against classifications by User 3.

(Model 1)	Blob	Tadpole	Donut	Arc	Other
Sensitivity	0.86	0.24	1	0.92	0.24
Specificity	0.96	0.98	0.99	0.84	0.98
Precision	0.97	0.49	0.39	0.67	0.31
F score	0.92	0.32	0.57	0.78	0.27

**Fig 7:**
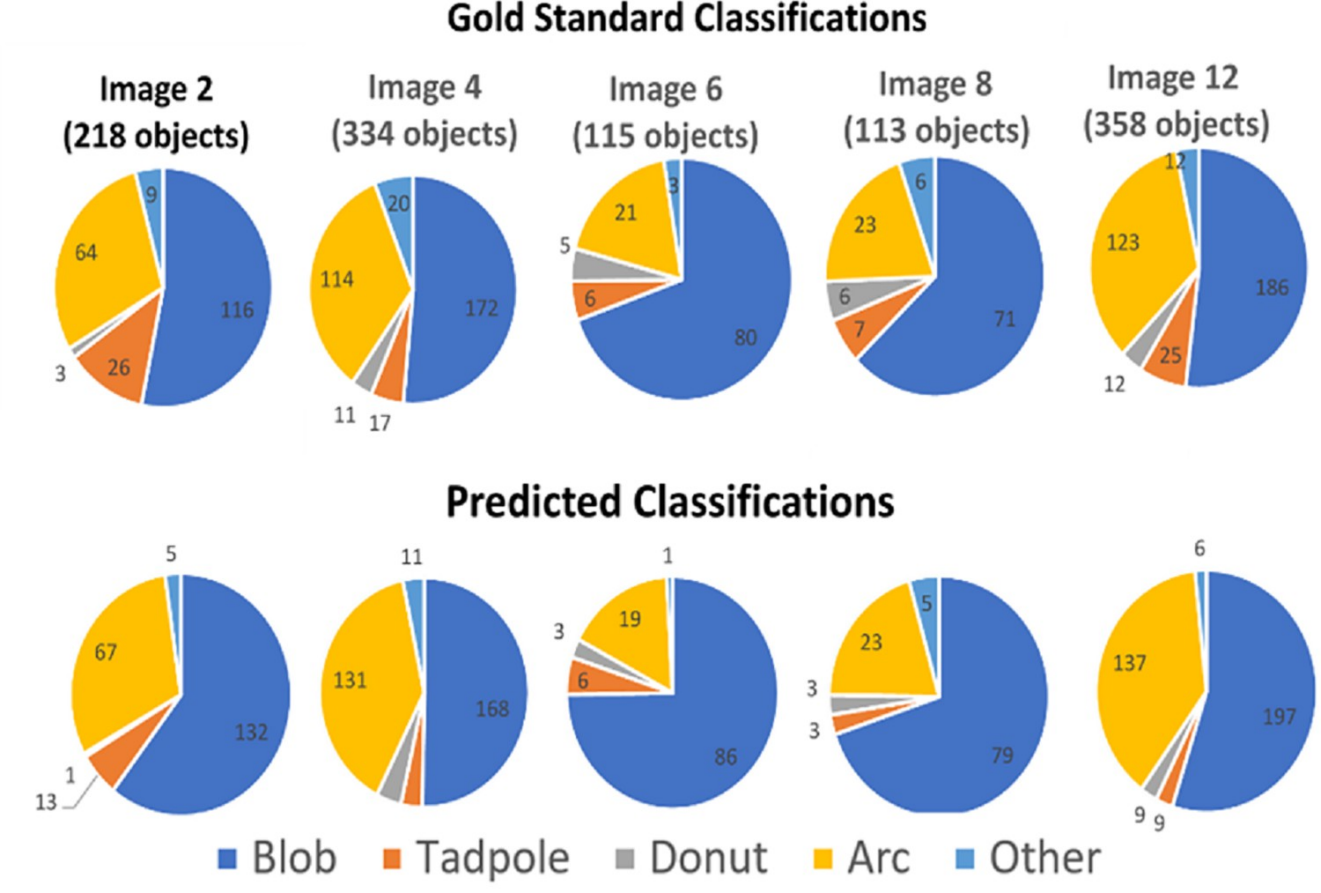
Class distributions for the initial 5 images. The gold standard classifications are in the top row and the predictions made by Model 2 are in the bottom row.

When the algorithm was trained using all 1,138 object classifications from the first 5 images (Model 2), the training accuracy, i.e. class prediction made using the training set was 84.8% with the performance breakdown per class as given in Table 5. The training performance remained similar to that when training with objects from one image. The breakdown per class is visually shown for the manual classifications and predicted classification in Fig 7.

**Table 5:** Training performance of model 2 against all gold standard labels.

(Model 2)	Blob	Tadpole	Donut	Arc	Other
Sensitivity	0.97	0.36	0.67	0.84	0.36
Specificity	0.89	0.99	0.99	0.89	0.99
Precision	0.91	0.69	0.86	0.77	0.64
F score	0.94	0.47	0.76	0.80	0.46

**Fig 8:**
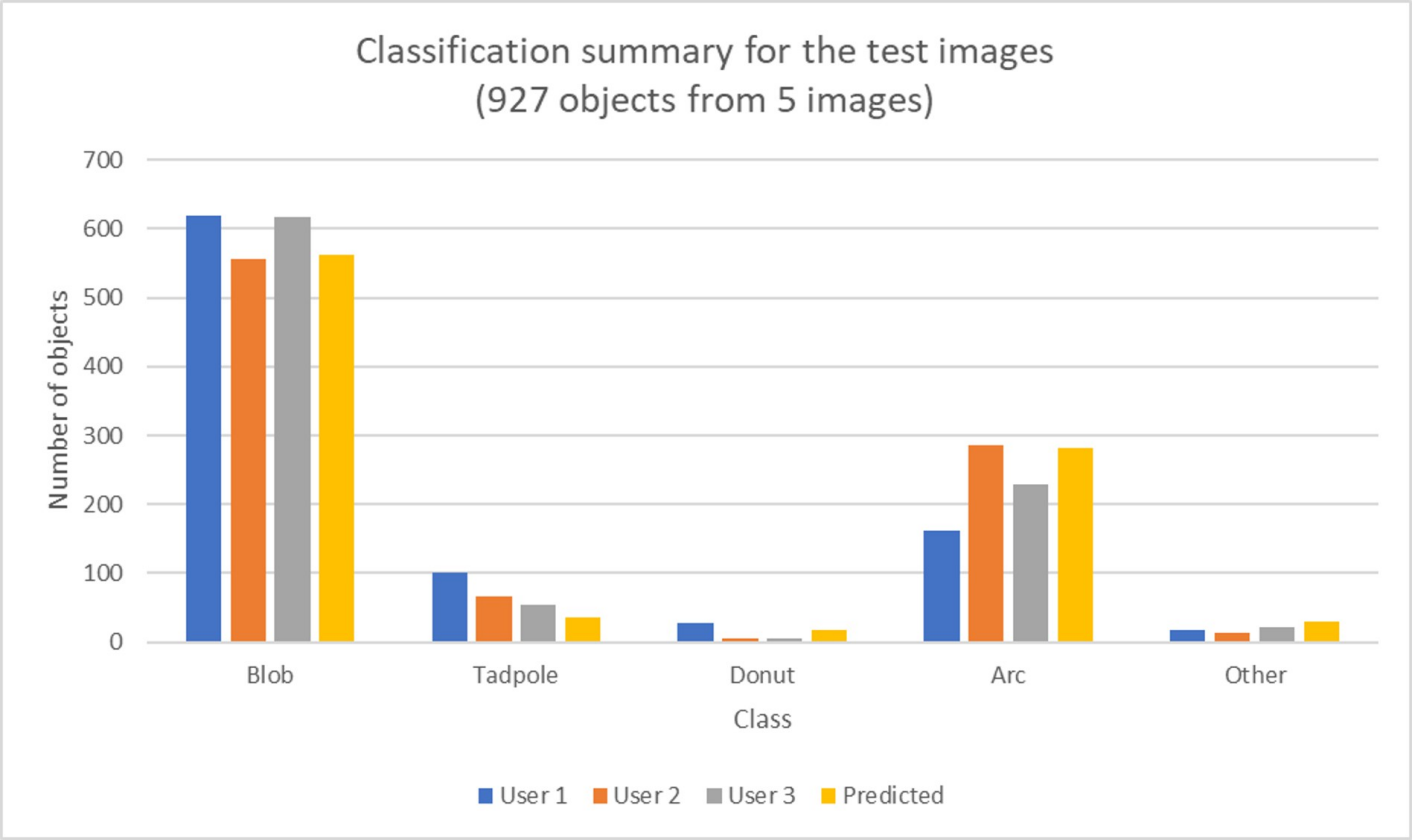
Summary of the number of objects each user classified as one of the five classes for the objects in the 5 test images along with the trained algorithm’s predicted classifications.

Testing Model 2 on the set of 5 new images that were classified individually produced an accuracy of 76.9%, 83.1%, and 85% for user 1, 2, and 3 respectively. A summary of the number of the total objects of each class that were identified by each user in this test set is shown in in Fig 8. The performance breakdown by user is given in Tables 6–8. The overall accuracy for each user increased and the performance for the Blob class remained high along with the overall high specificity for all classes.

**Table 6:** Testing performance of model 2 against User 1 classifications.

(Model 2)	Blob	Tadpole	Donut	Arc	Other
Sensitivity	0.86	0.27	0.37	0.85	0.29
Specificity	0.90	0.99	0.99	0.81	0.97
Precision	0.95	0.75	0.55	0.49	0.17
F score	0.905	0.39	0.44	0.62	0.28

**Table 7:** Testing performance of model 2 against User 2 classifications.

(Model 2)	Blob	Tadpole	Donut	Arc	Other
Sensitivity	0.92	0.43	0.80	0.77	0.38
Specificity	0.86	0.99	0.98	0.91	0.97
Precision	0.92	0.80	0.22	0.79	0.17
F score	0.91	0.56	0.35	0.78	0.24

**Table 8:** Testing performance of model 2 against User 3 classifications.

(Model 2)	Blob	Tadpole	Donut	Arc	Other
Sensitivity	0.89	0.52	0.83	0.83	0.32
Specificity	0.95	0.99	0.98	0.88	0.97
Precision	0.97	0.78	0.28	0.71	0.24
F score	0.93	0.62	0.43	0.78	0.27

In the final validation step 3, a total of 438 objects were collectively validated by one of the three users, of which the same 24 objects were randomly selected by two users for validation. These results are summarized in Table 9 and showed 87% of the validated objects were accurately predicted. A breakdown per class of the validated objects’ predicted class to manual classification is presented in Table 10. Looking at each user individually: user 1 validated 173 objects indicating 89% were correct, user 2 validated 122 objects indicating 88% were correct, and user 3 validated 167 objects indicating 86% were correct.

**Table 9:** Summary of validation of classifications.

	validated	correct	incorrect	%correct	%incorrect
All (excl duplicates)	438	381	57	86.98	13.01
All (incl duplicates)	462	404	58	87.44	12.55

**Table 10:** Breakdown of validation by class.

	Manual classification (excluding duplicates)					
%error (of 13%)	Predicted class	Blob	Tadpole	Donut	Arc	Other
5	Blob	[209]	5	11	3	3
1.6	Tadpole	1	[19]	0	6	0
0	Donut	0	0	[14]	0	0
4.3	Arc	5	9	0	[125]	5
2.1	Other	0	2	3	4	[14]

## Discussion

The motivation for the development of the approach described here was to investigate the mitochondrial morphology to better understand the metabolic activity and replication potential of *T*. *gondii* bradyzoites. However, the approach can serve as a template for developing automated classification of morphologies of a variety of sub-cellular organelles where there is some ambiguity and potential for variation and lack of reproducibility in manual classification due to inherent biological diversity and operator fatigue. The advantage of automated approaches of classification are the ability for processing large numbers of objects. In addition, in situations where there is ambiguity in manual classification [13], automated approaches trained using consensus classification can be advantageous as they apply consistent logic for classification and thus minimize the potential for variation and bias.

*T*. *gondii* bradyzoites within tissue cysts have been found to be metabolically active with heterogeneous replication [2]. Therefore, classification of mitochondrial morphologies that are present within the bradyzoites can inform about the metabolic state of the bradyzoite as a functional state of any given cell is likely to be associated with the particular morphology of the mitochondrion [5, 6]. Image processing and classification algorithms can facilitate the analyses of larger numbers of images than what would be possible by the current tedious and time-consuming process of manually classifying the mitochondria from the thousands of possible bradyzoites within a cyst. This increased throughput will allow the gathering of information about the biology of this parasite at a scale that is not possible using current approaches.

The developed program helped to facilitate the manual classification of mitochondrial shapes, obtaining consensus classification, and these classifications were used in the evaluation of a machine learning approach for the automation. A common machine learning method used in object detection for multi-class classification is multinomial logistic regression approach with a one vs all methodology [18], which was used in this study to predict the detected objects’ morphological class. With the extracted features and user labeled data collected via the developed program, this approach proved to be successful for the mitochondria morphology classification problem presented here, with achieving up to 85% overall accuracy, i.e., the fraction of correctly classified objects regardless of class.

Blob morphology (condensed or punctate mitochondrial profile) was the simplest to classify with a readily apparent appearance which likely resulted in the high level of congruency among users and the higher accuracy that was also seen for the trained machine learning algorithm. Most of the objects were identified as belonging to the Blob morphology, with lower incidences of Tadpole and Arc morphologies which was not surprising as the Tadpoles and Arcs are associated with more active parasites and bradyzoites’ metabolic activity was expected to be low [5]. When training the algorithm with gold standard classifications of objects from one image compared with that of five images, there was a slight decrease in training accuracy but the testing accuracies slightly increased. With the larger training set, the model was better generalized to fit the new test data but these differences in accuracy were minimal, suggesting objects from one image may be sufficient for training but that more may be somewhat helpful. The trained machine learning performance resulted in a high degree of confidence in the prediction of Blobs and Arcs which make up a majority of morphological classes present in most bradyzoites. It is noteworthy that in the final validation step, the prediction accuracy was determined from a testing set which as much larger and de-novo from the training set, which mimics real-world use for these types of machine learning based automated classification approaches.

## Conclusion

Results of our study suggest that a computerized approach can be used to successfully automate the classification of the majority of mitochondria morphologies within encysted bradyzoites which can minimize the burden of manually evaluating these images and present opportunities for hybrid workflows involving partial automation. A hybrid workflow would automatically classify objects that were predicted to belong to a class with higher accuracy and where there was overall high congruency, such as seen with the Blobs. The classes which attained lower accuracy and which were also of low incidence could be presented to the user for manual classification, thus dramatically reducing the time required for manual classification.

## Supporting information

S1 Raw images (PDF)

S2 Raw images (PDF)

S3 Raw images (PDF)

S4 Raw images (PDF)

S5 Raw images (PDF)

S6 Raw images (PDF)
