# Supplementary figures and images for: Machine learning based classification of mitochondrial morphologies from fluorescence microscopy images of Toxoplasma gondii cysts
Source: PLoS One. 2023 Feb 2;18(2):e0280746. doi: 10.1371/journal.pone.0280746 (PMC9894464; doi:10.1371/journal.pone.0280746)

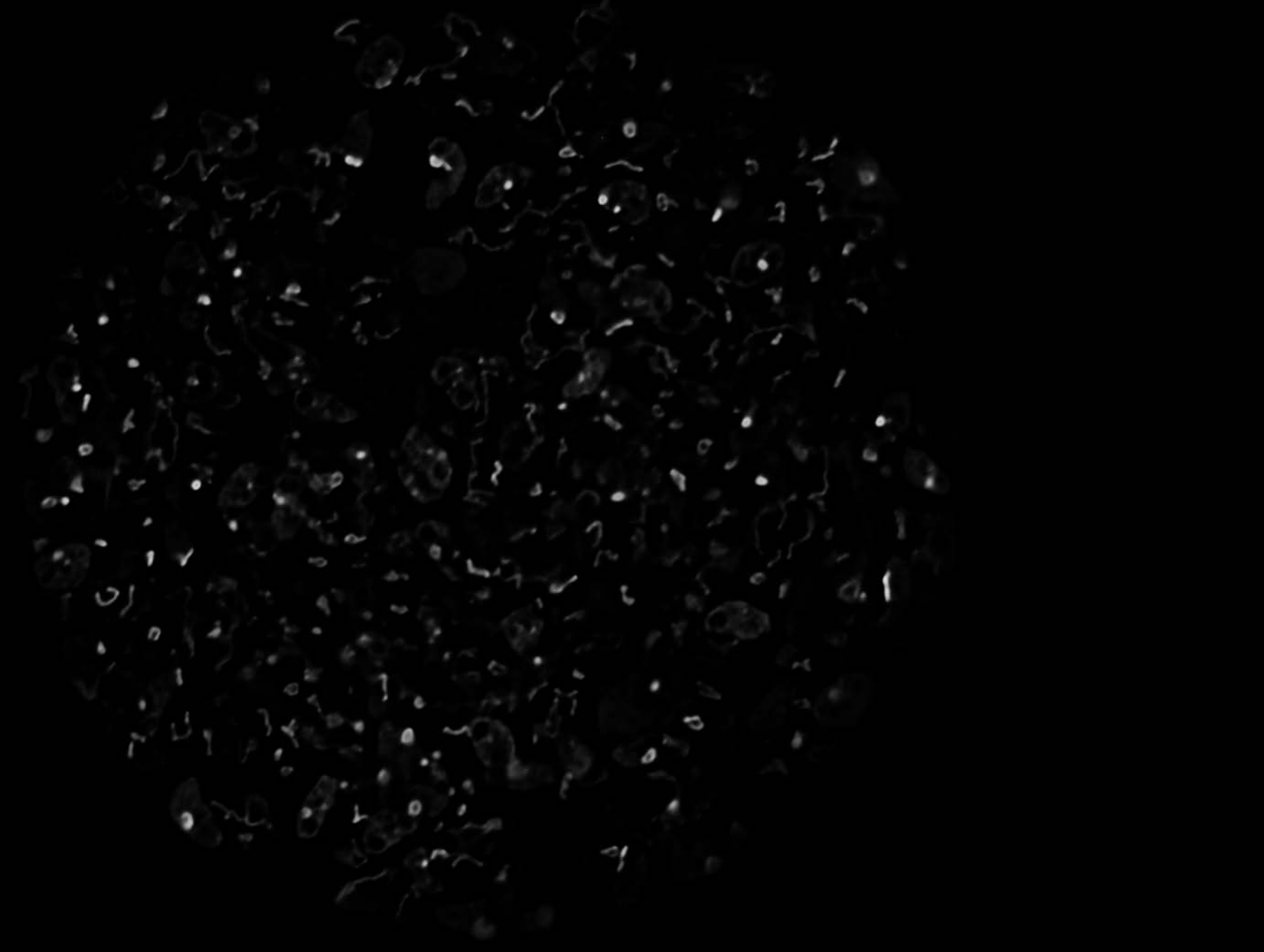

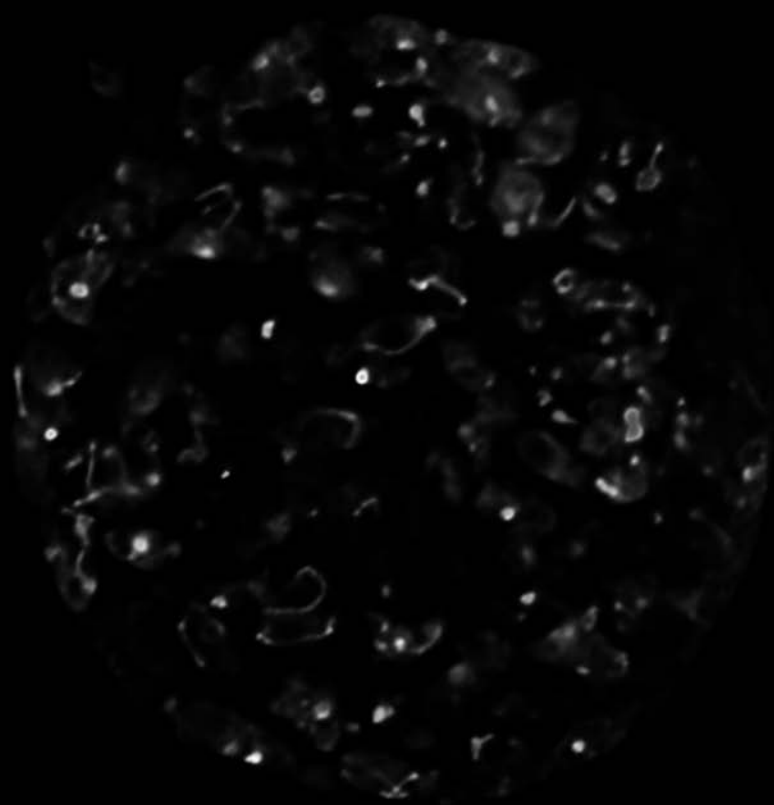

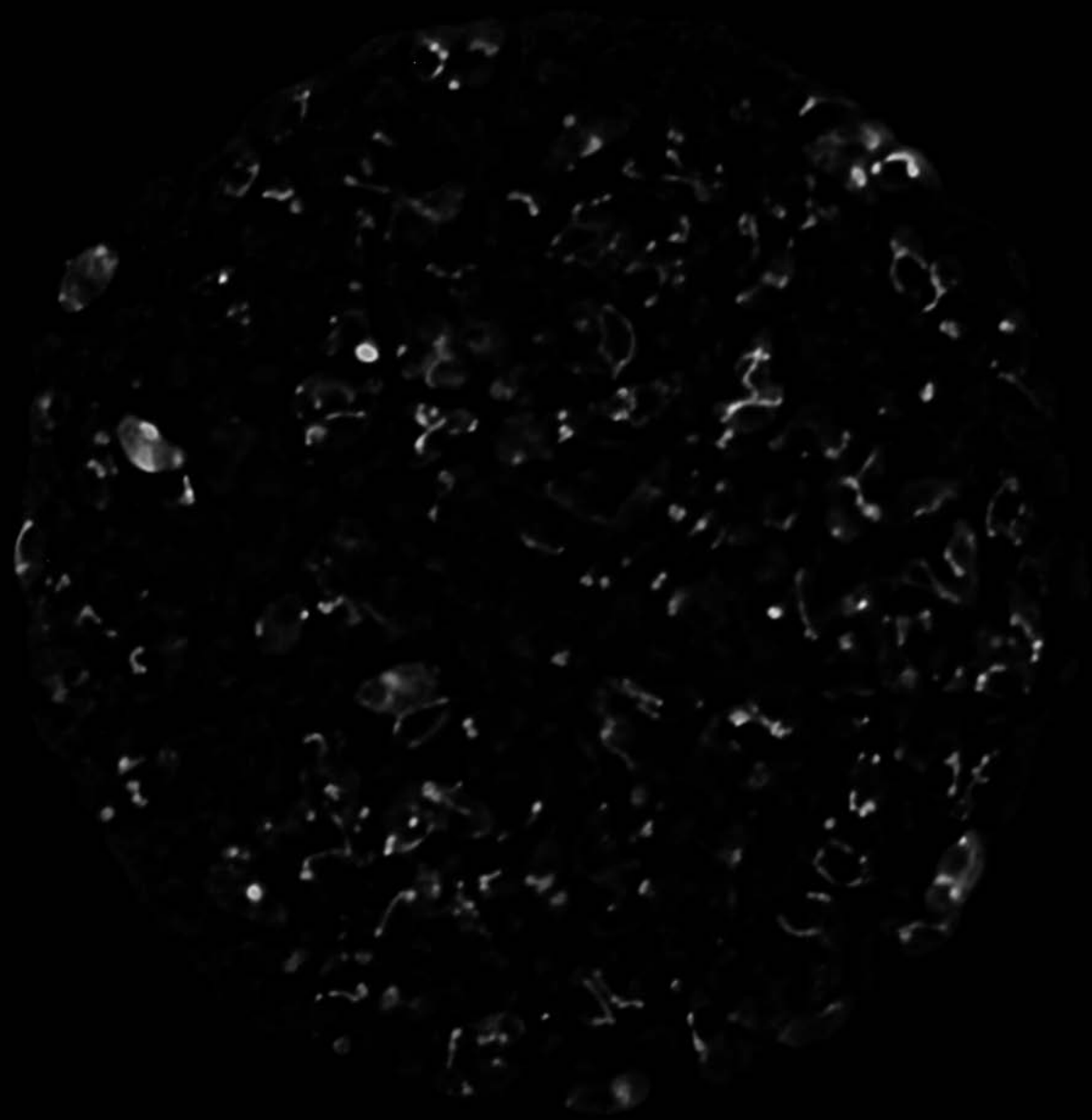

Supplement: S1 Raw images — (PDF) [file pone.0280746.s001.pdf]

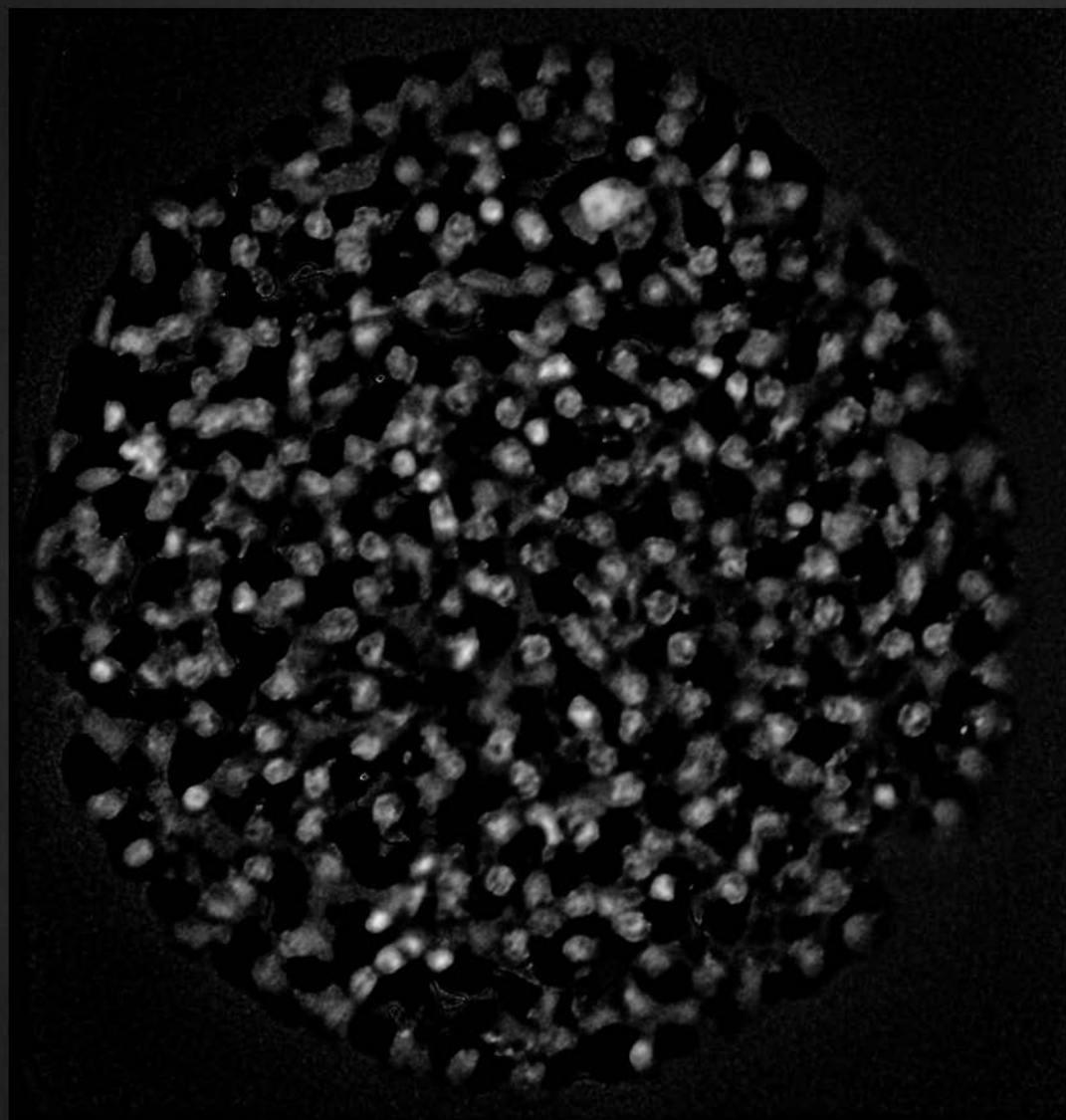

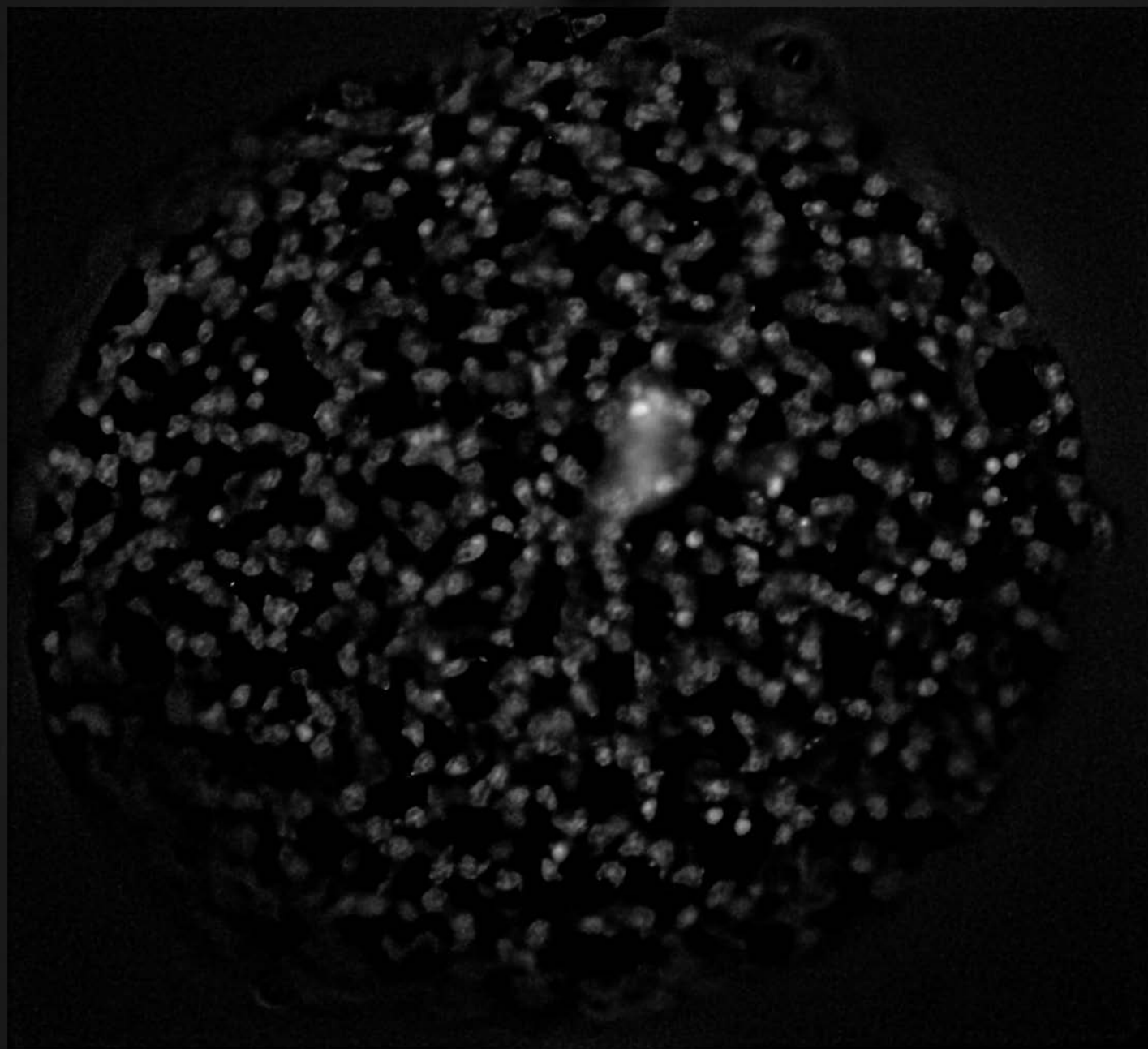

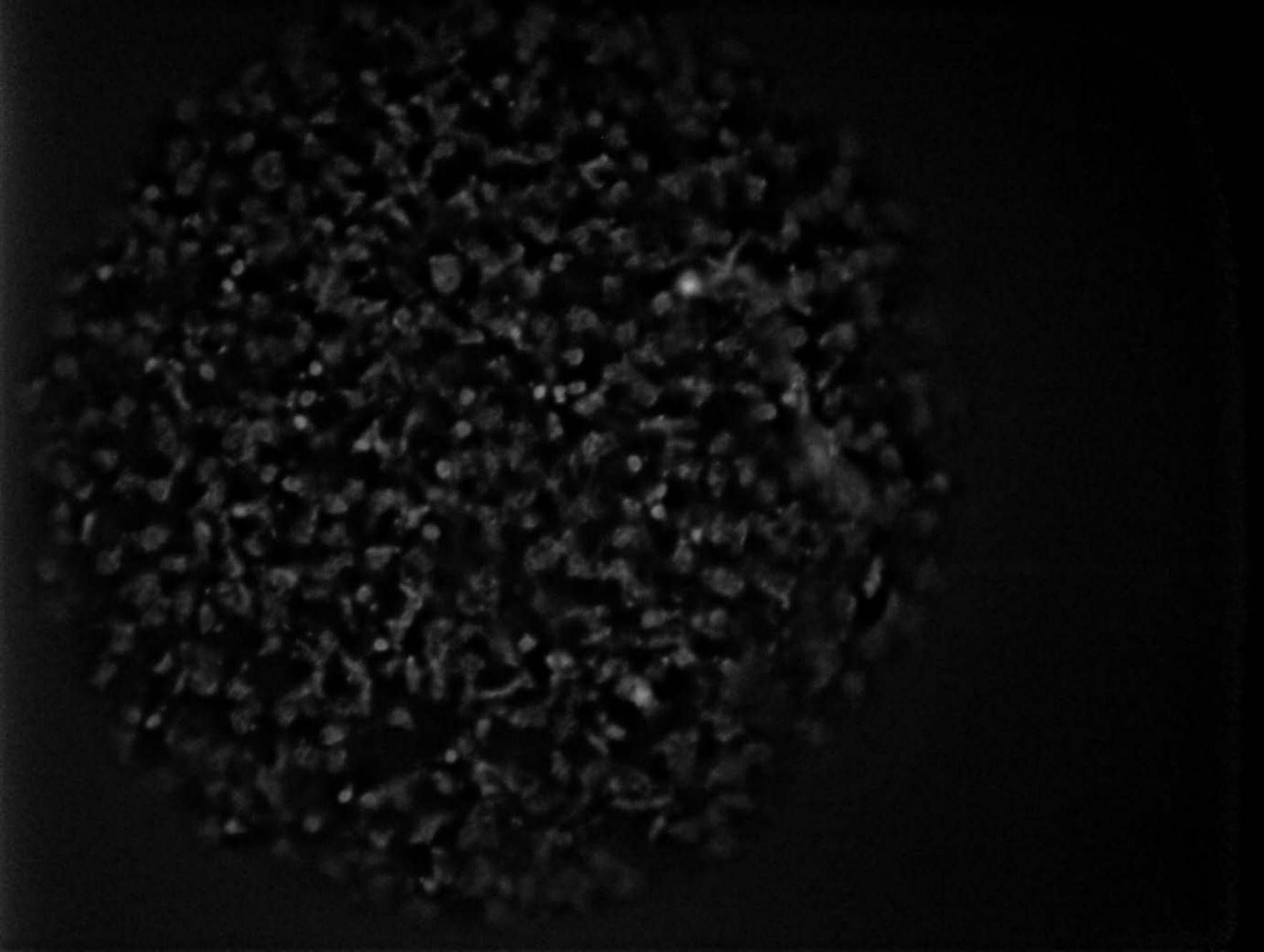

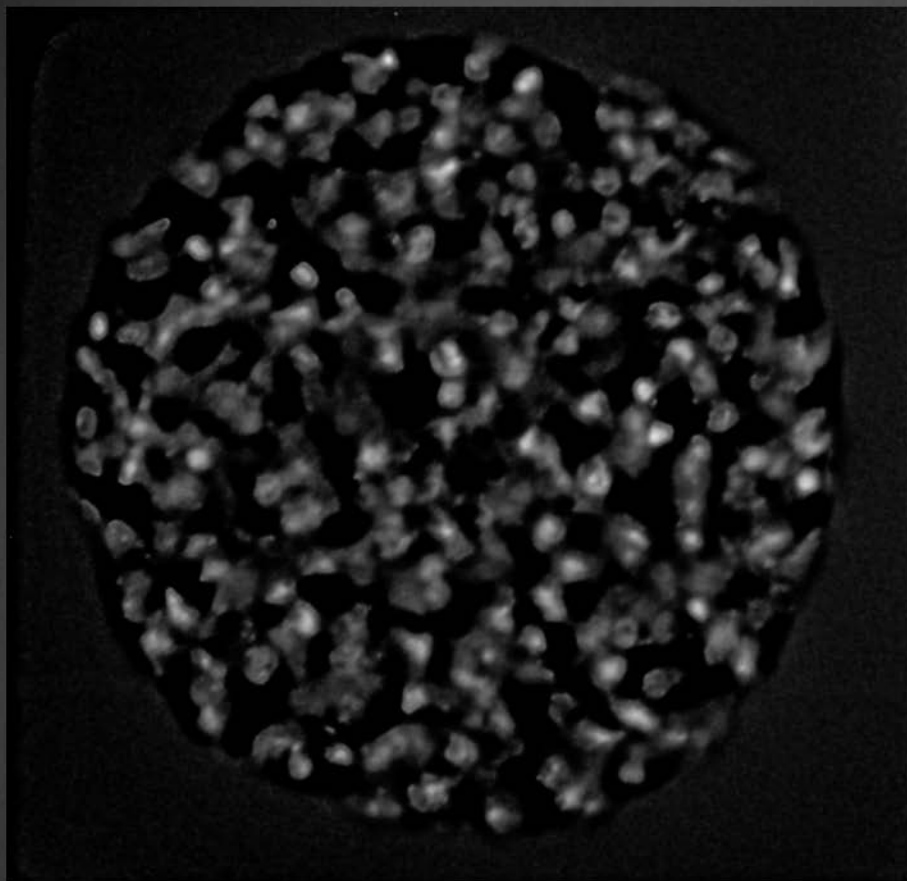

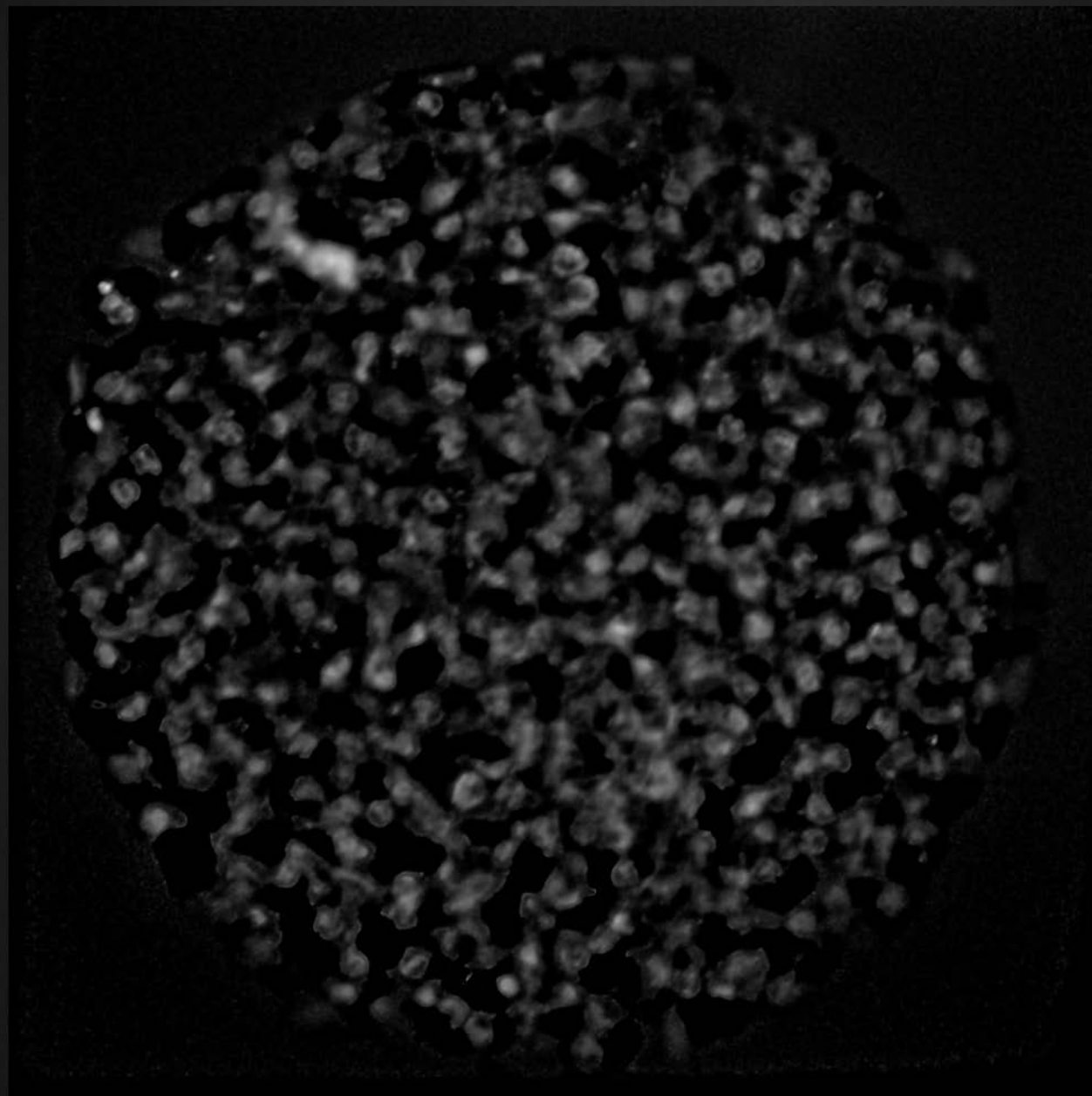

Supplement: S2 Raw images — (PDF) [file pone.0280746.s002.pdf]

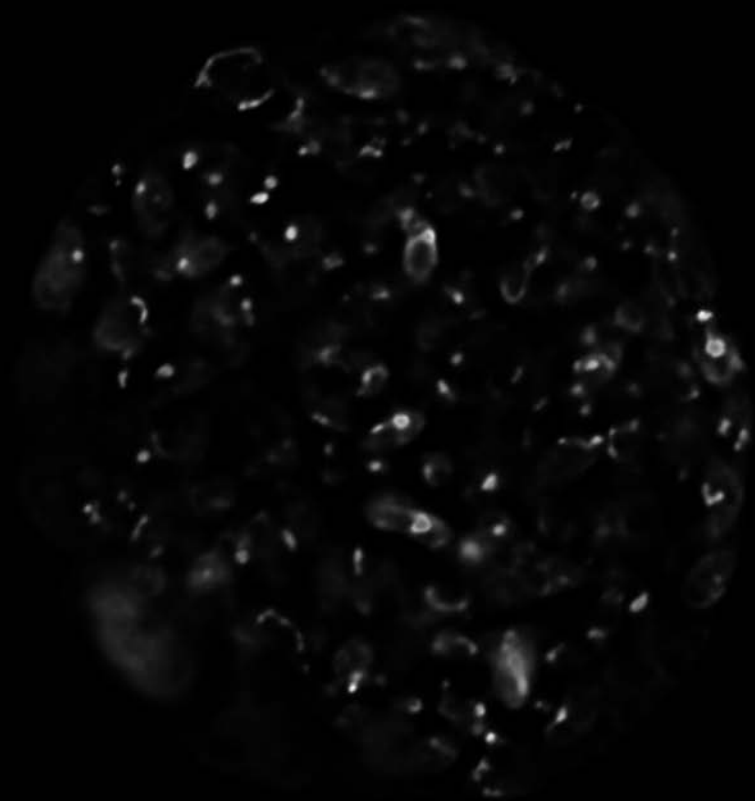

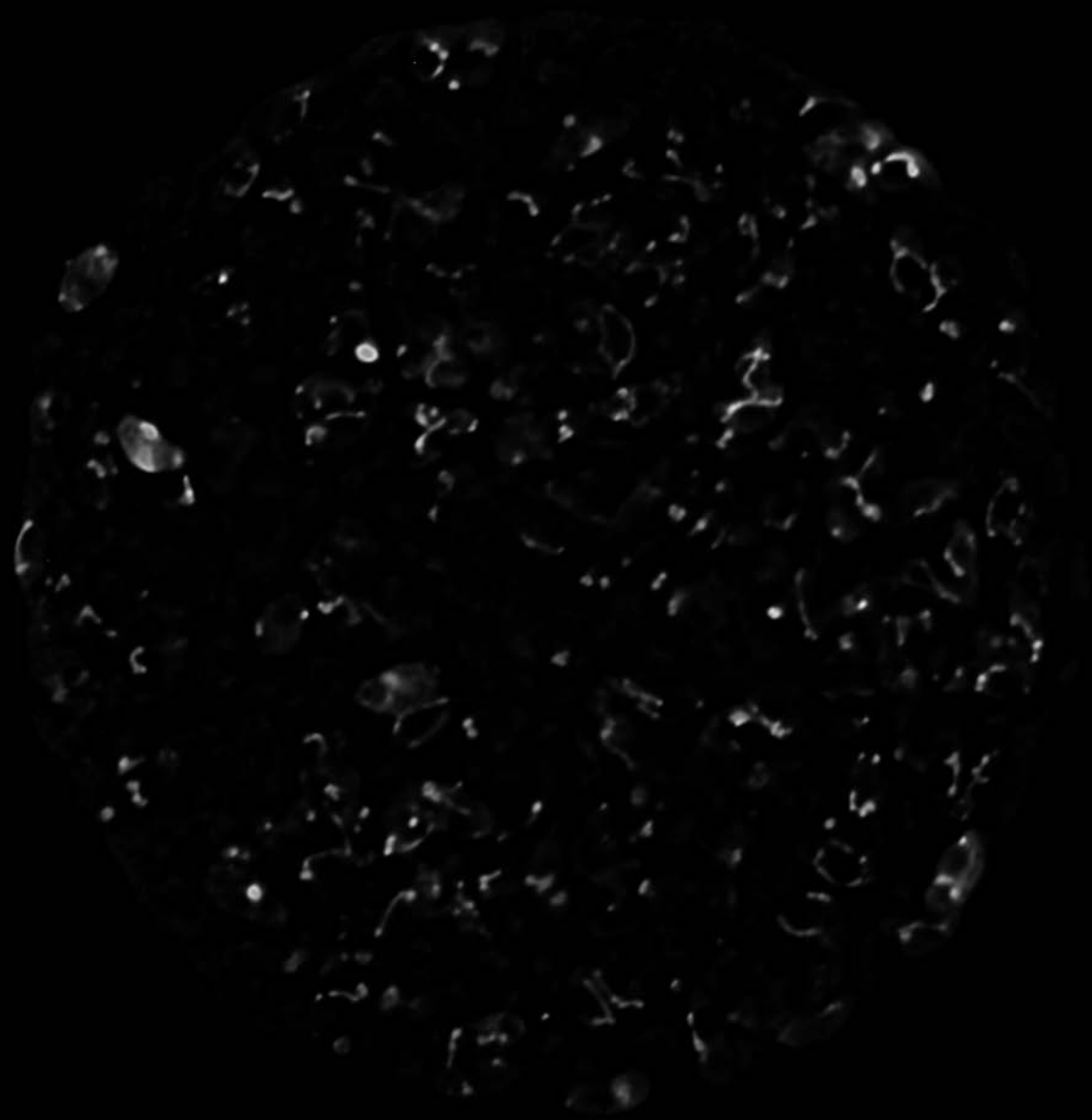

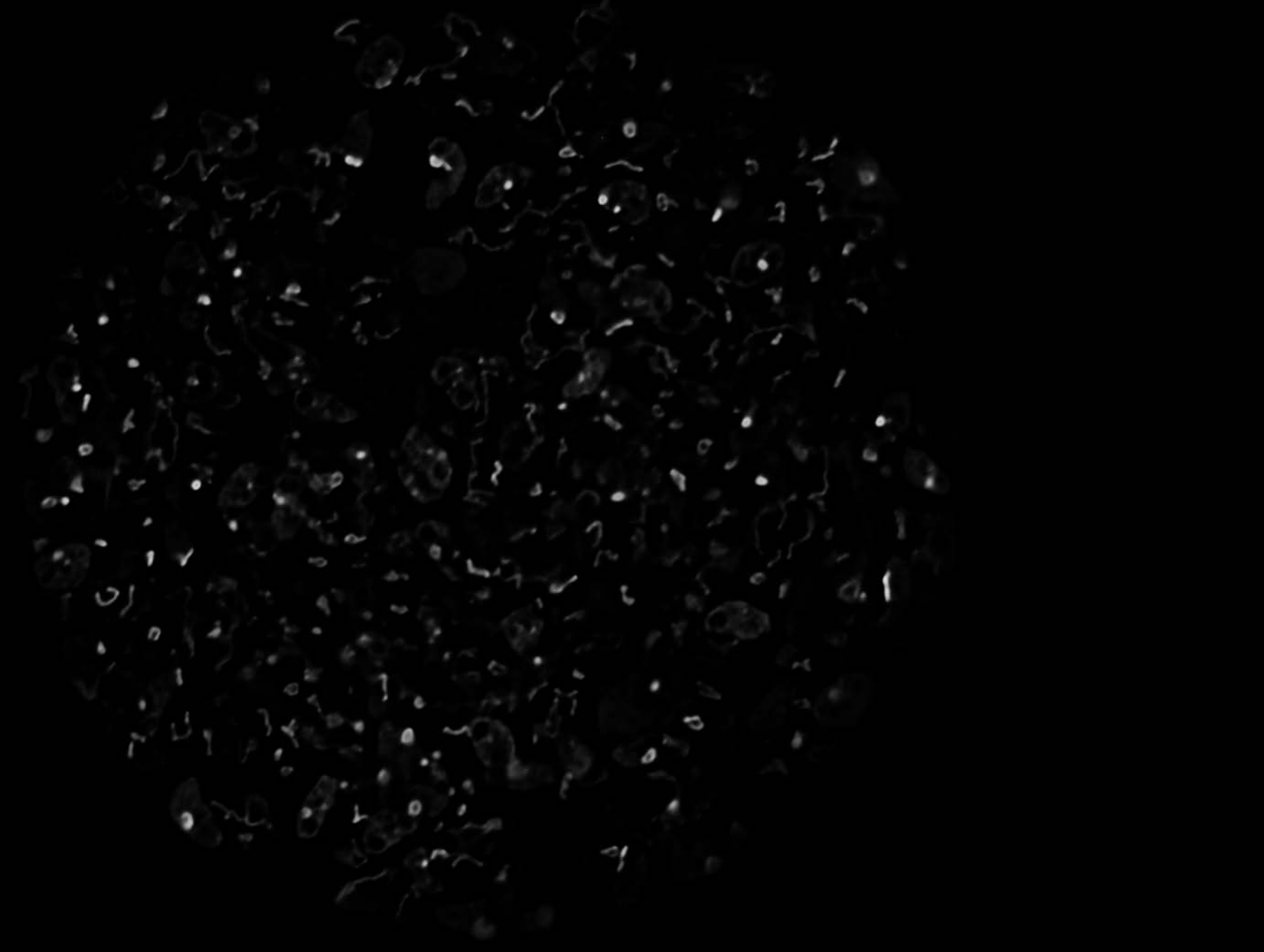

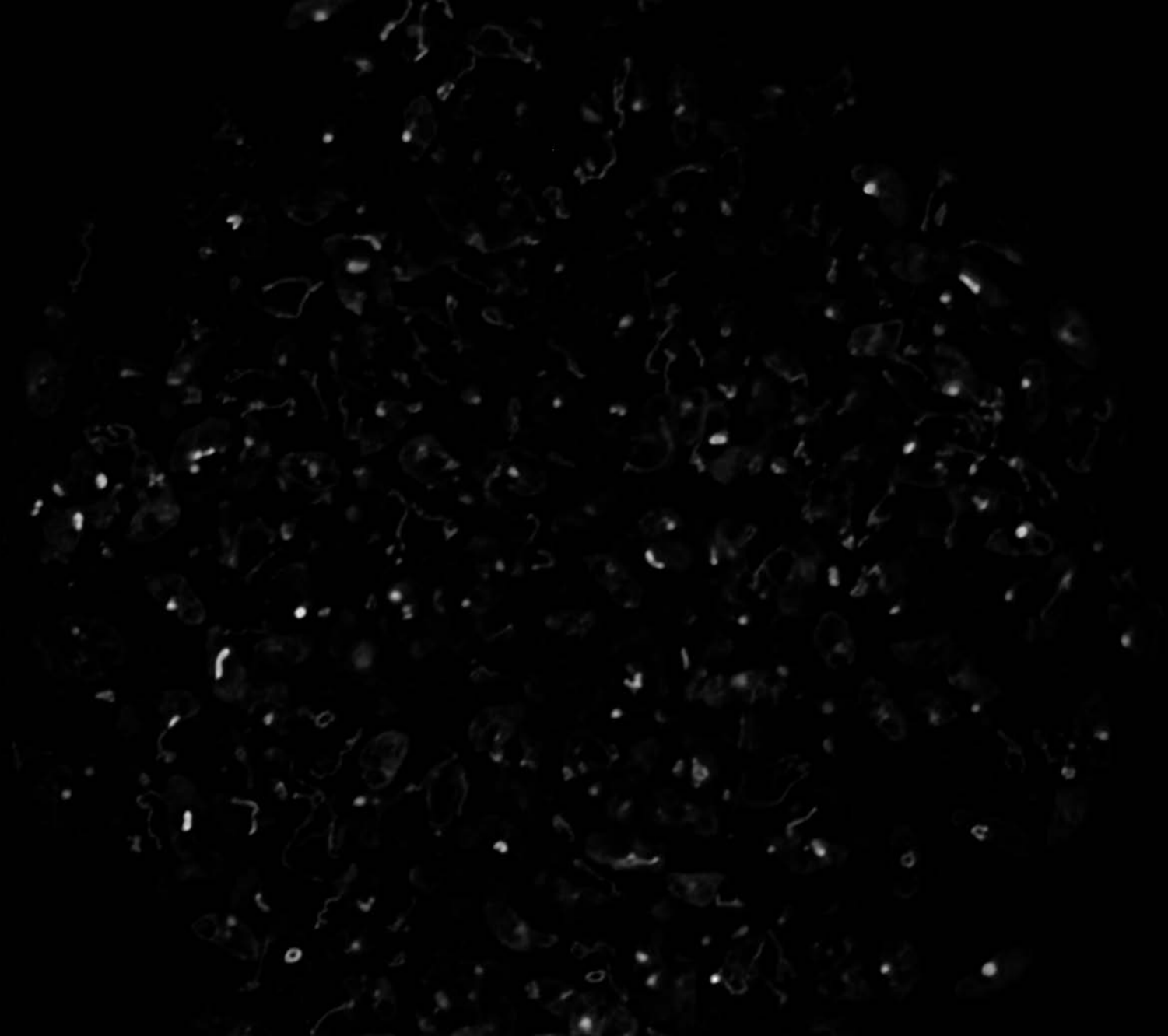







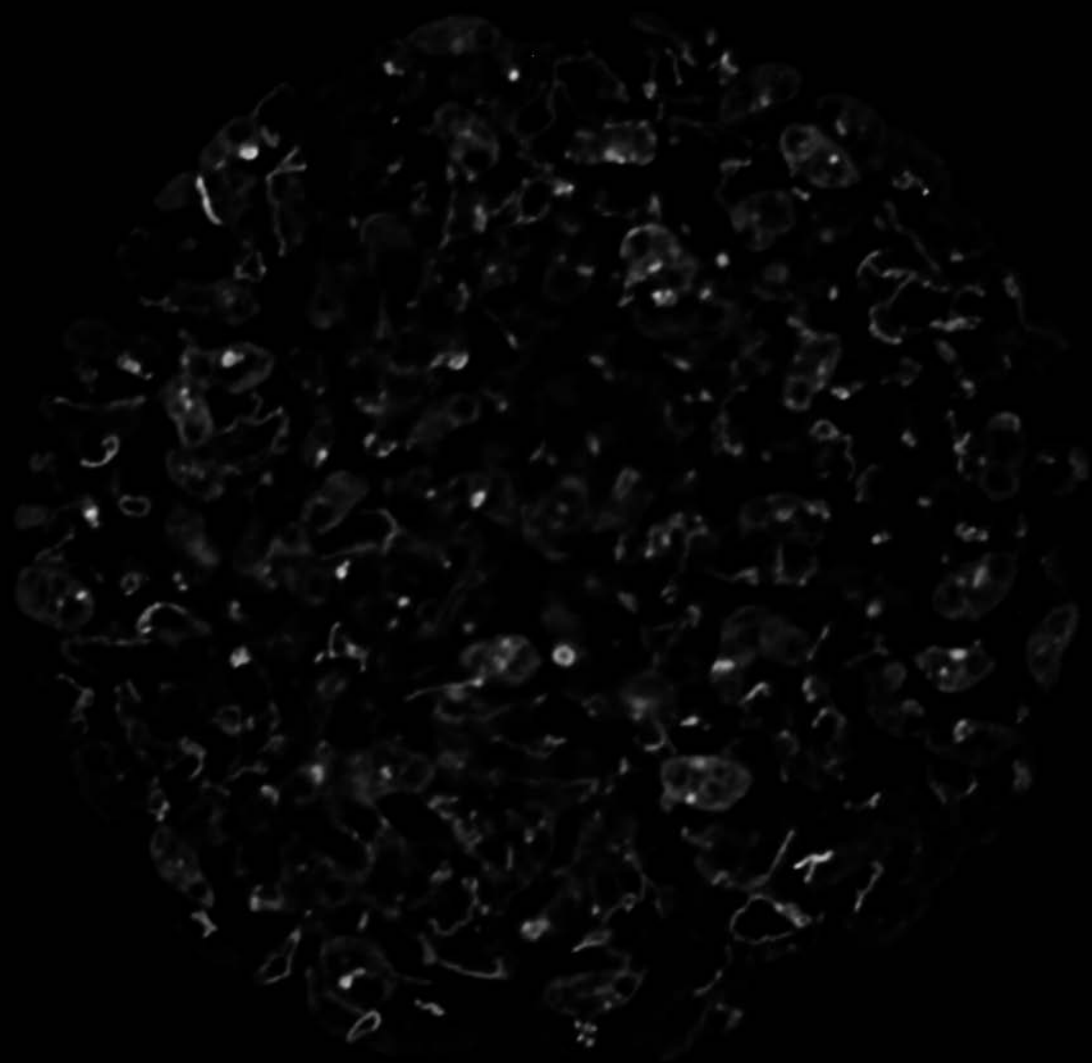



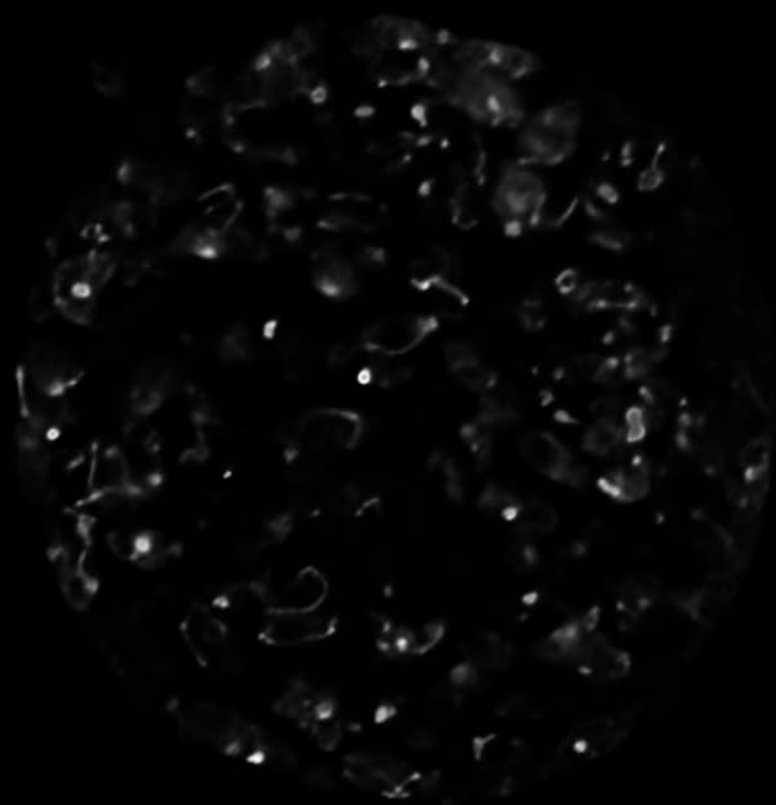

Supplement: S3 Raw images — (PDF) [file pone.0280746.s003.pdf]

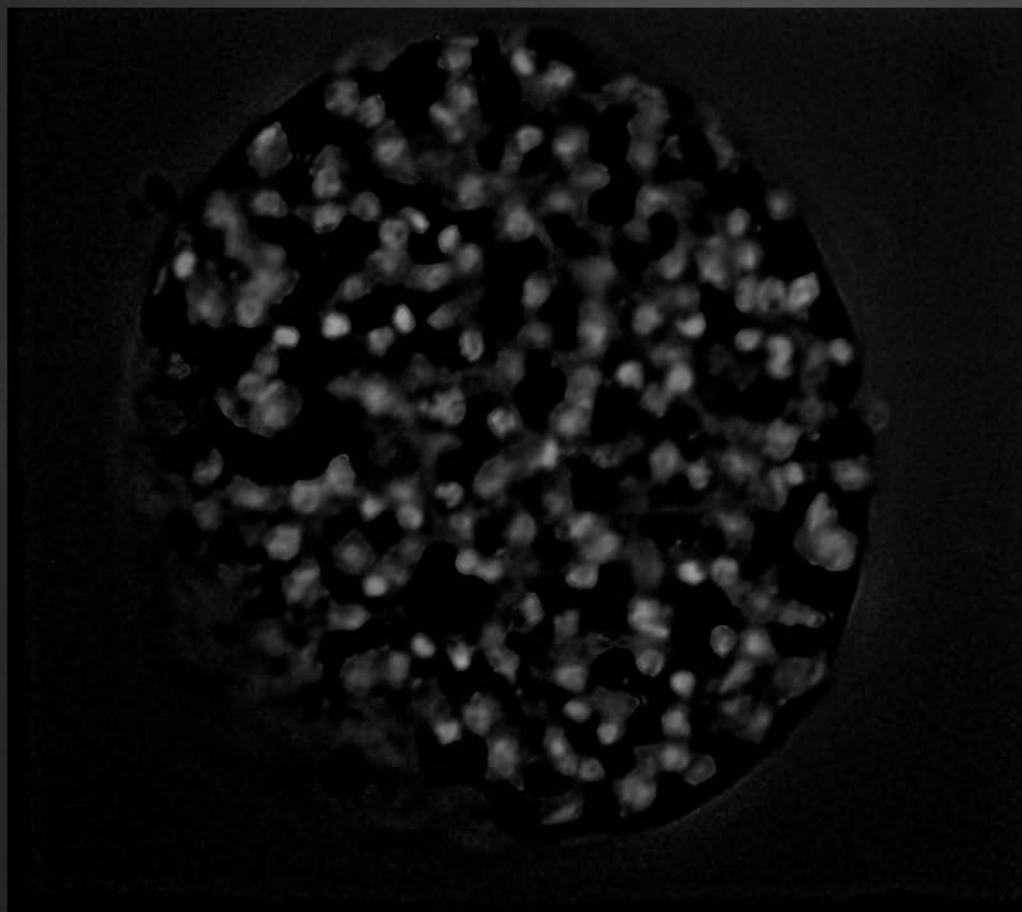

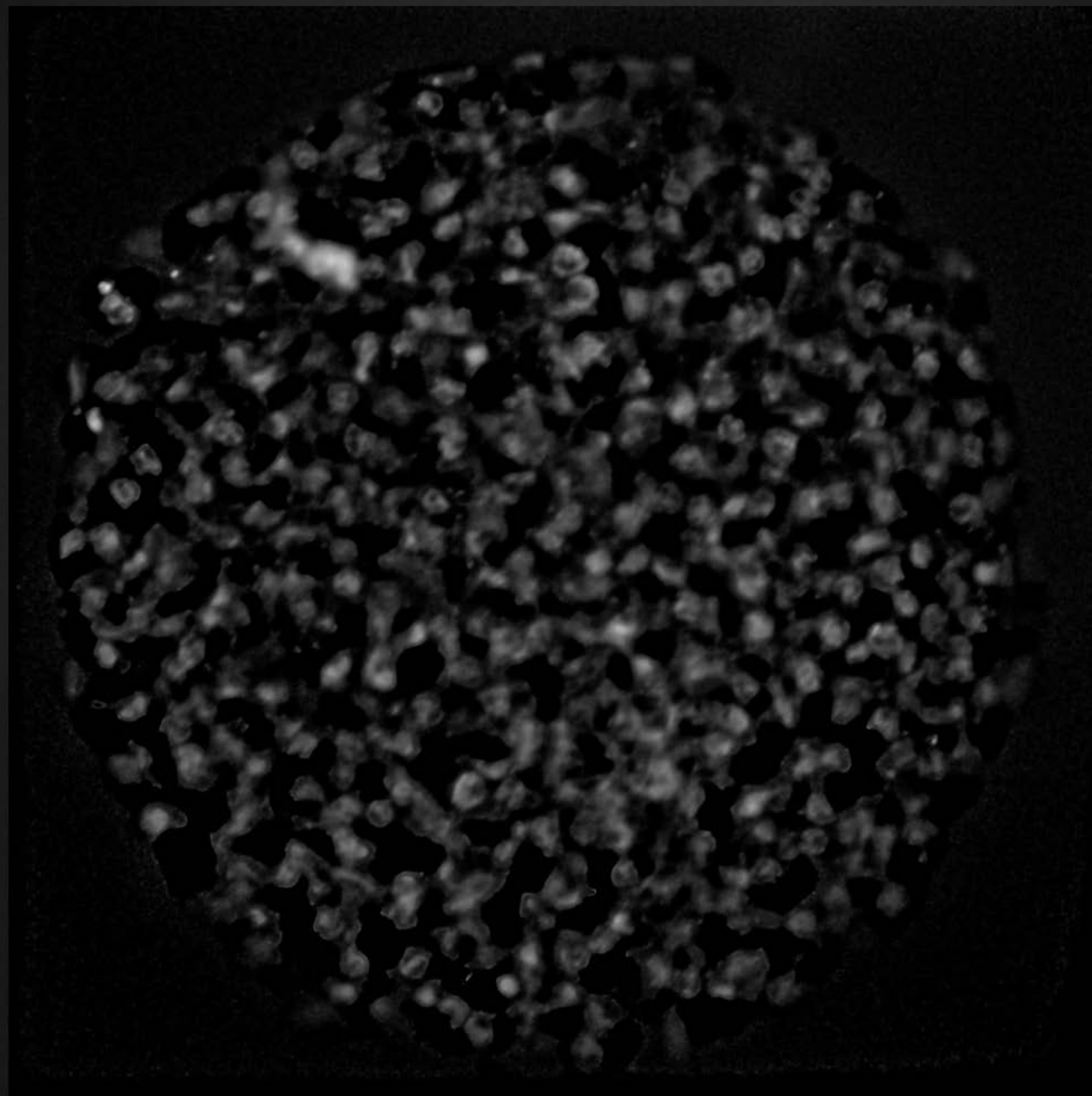

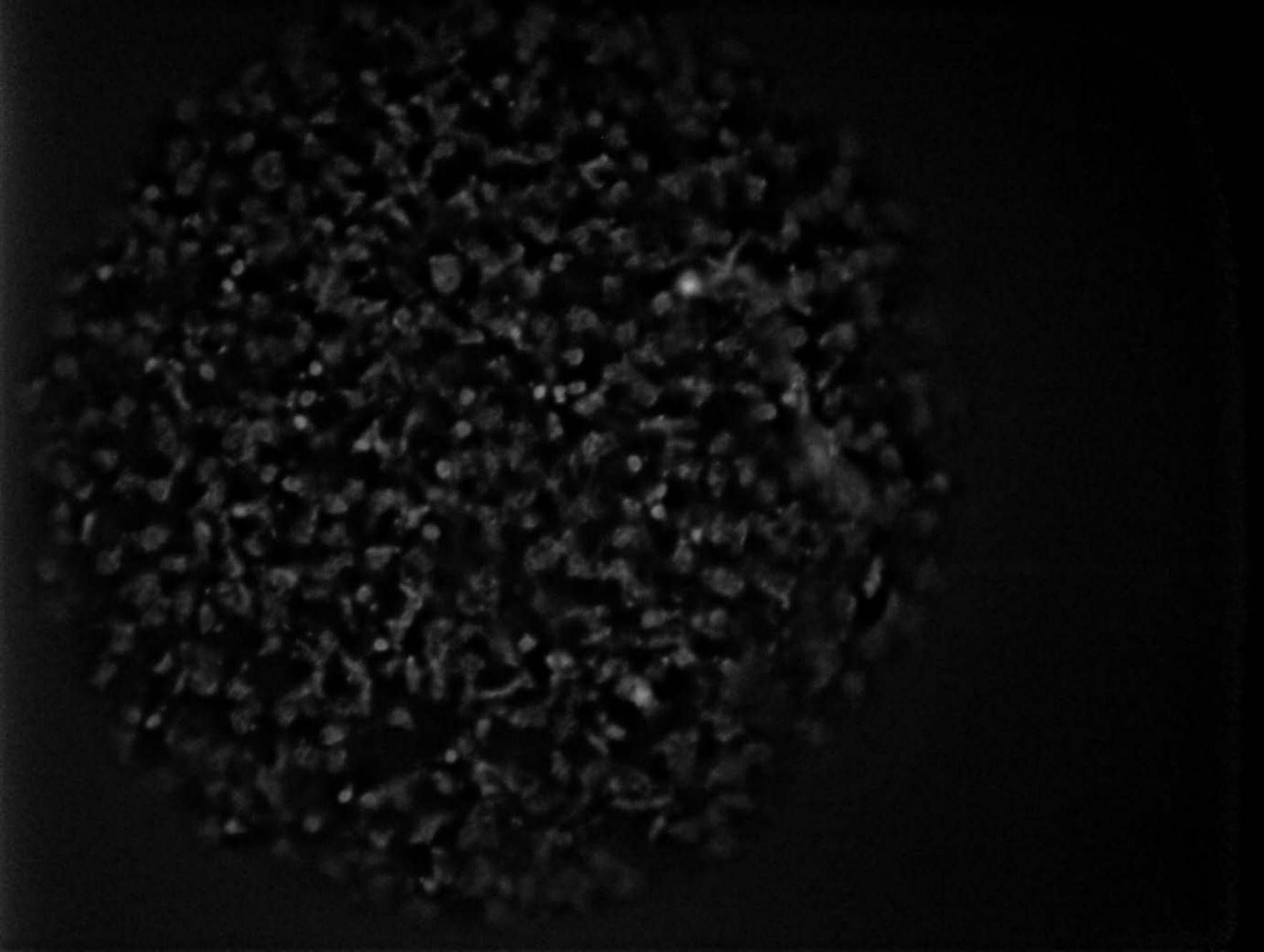

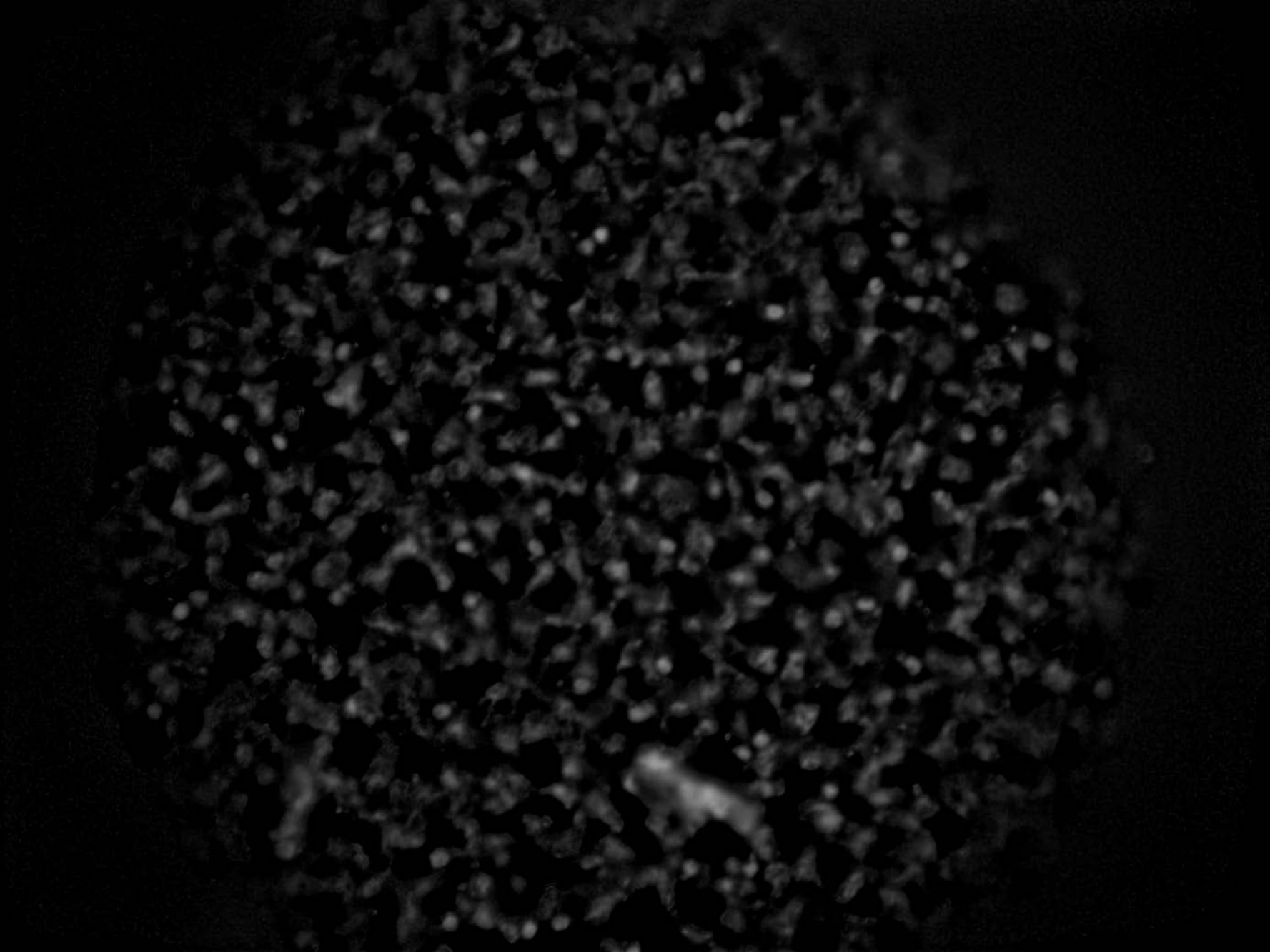

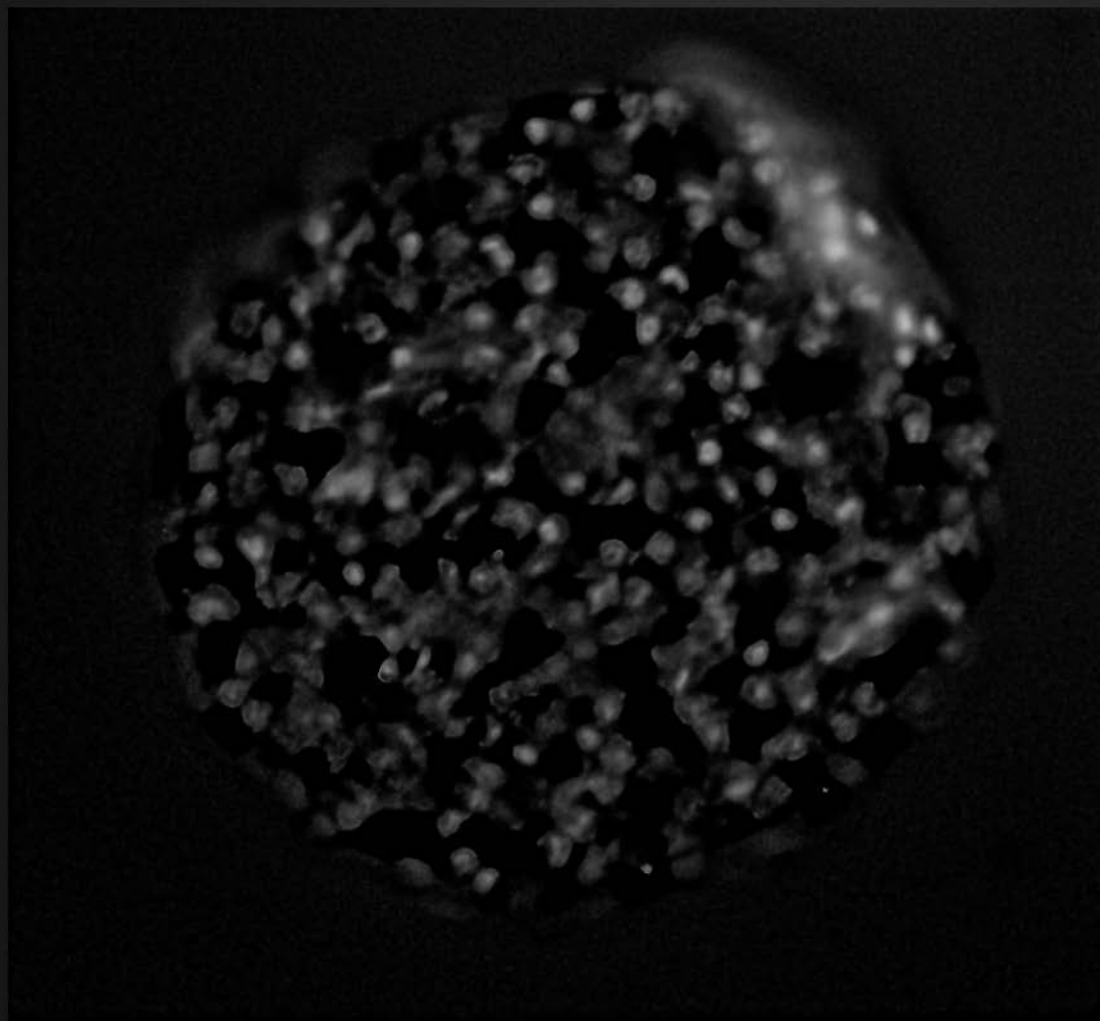

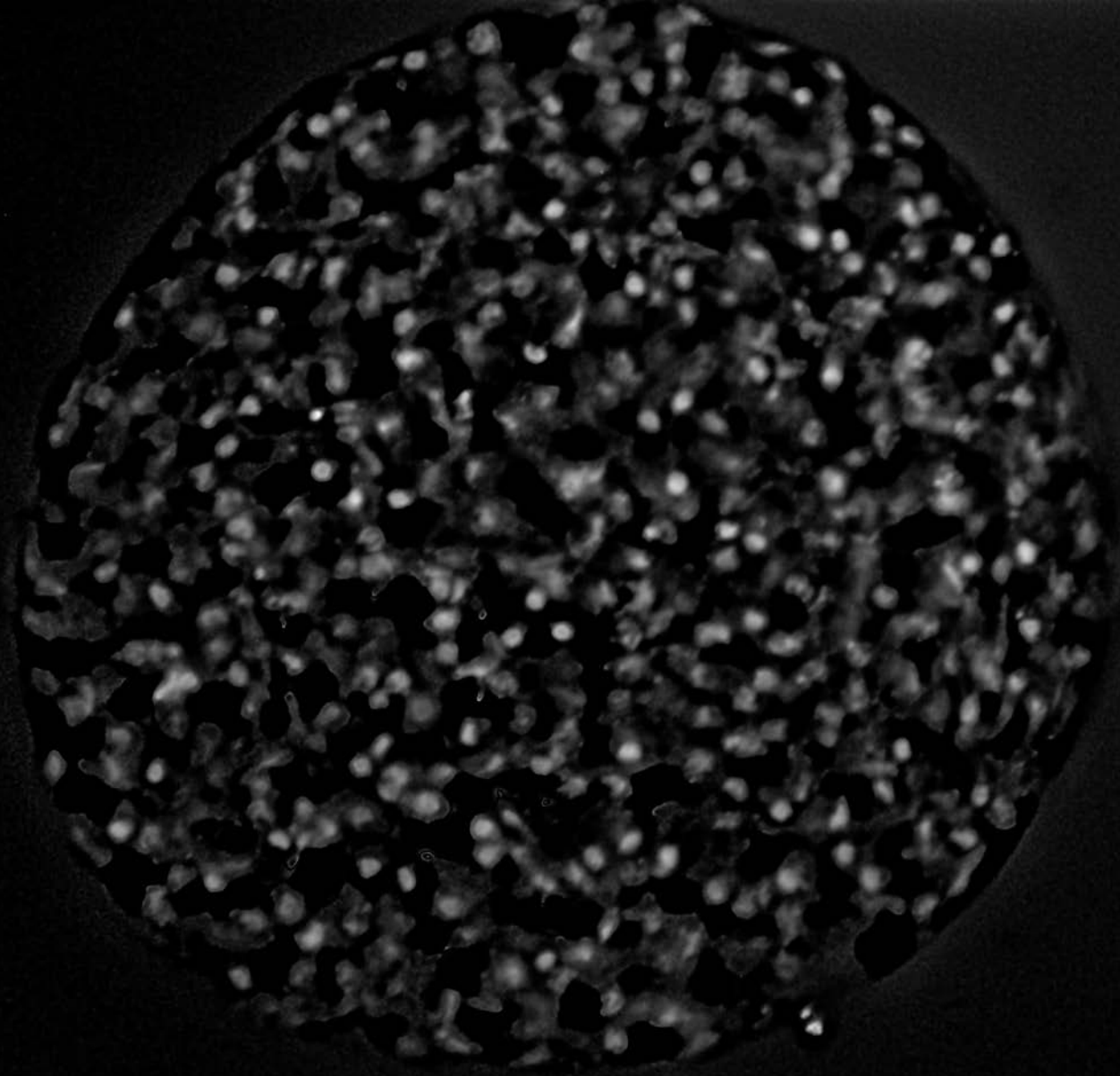

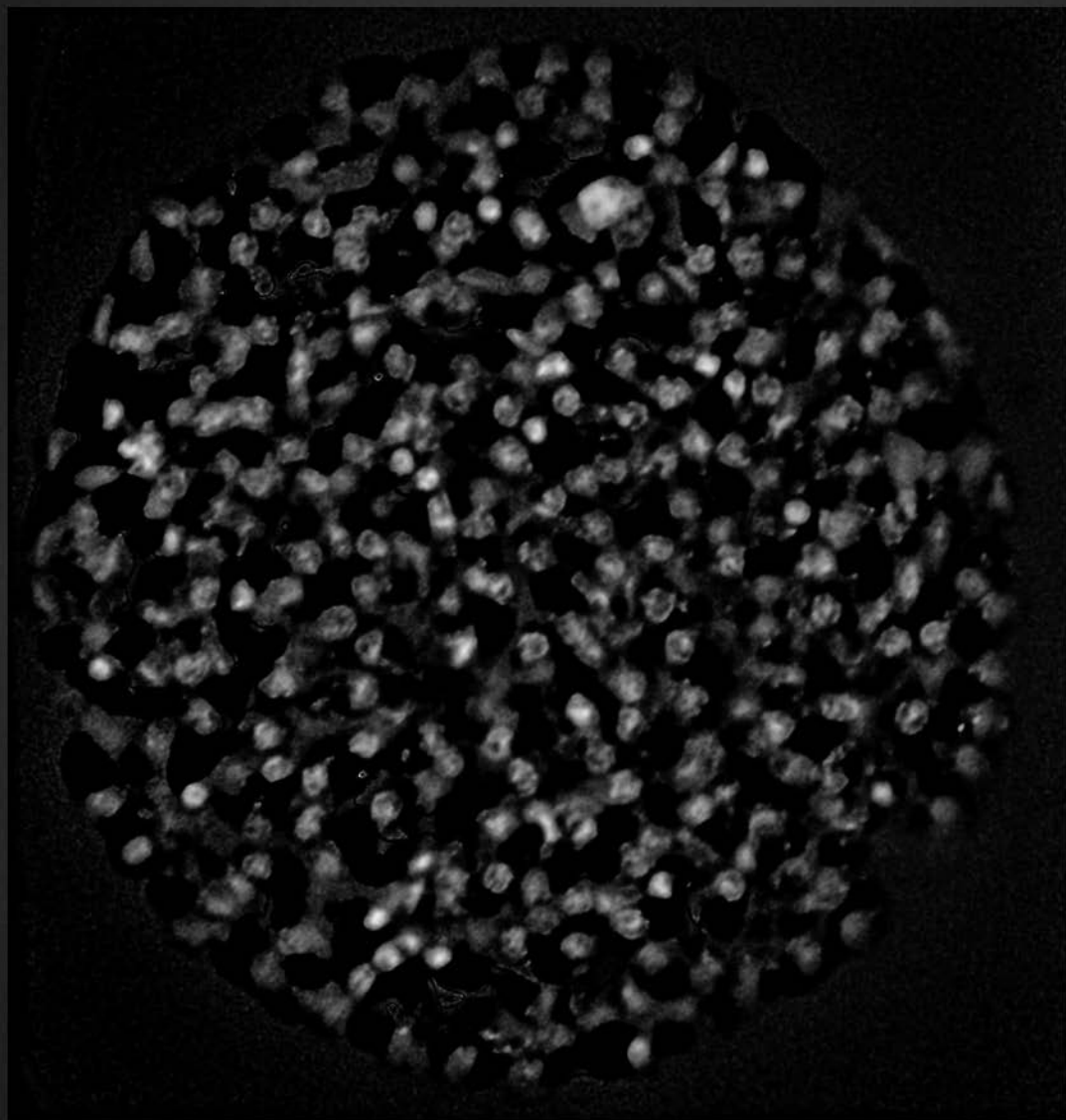

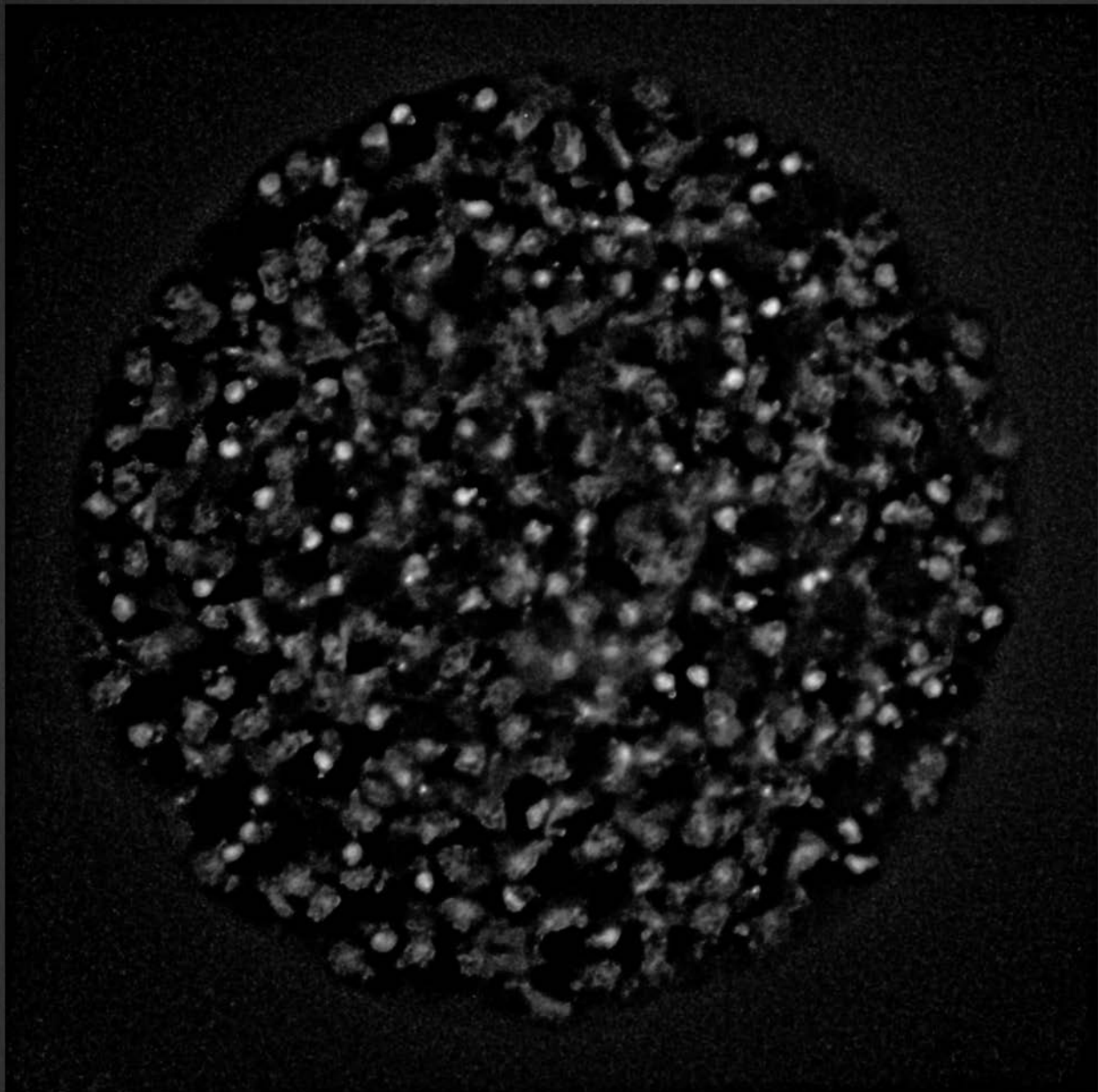

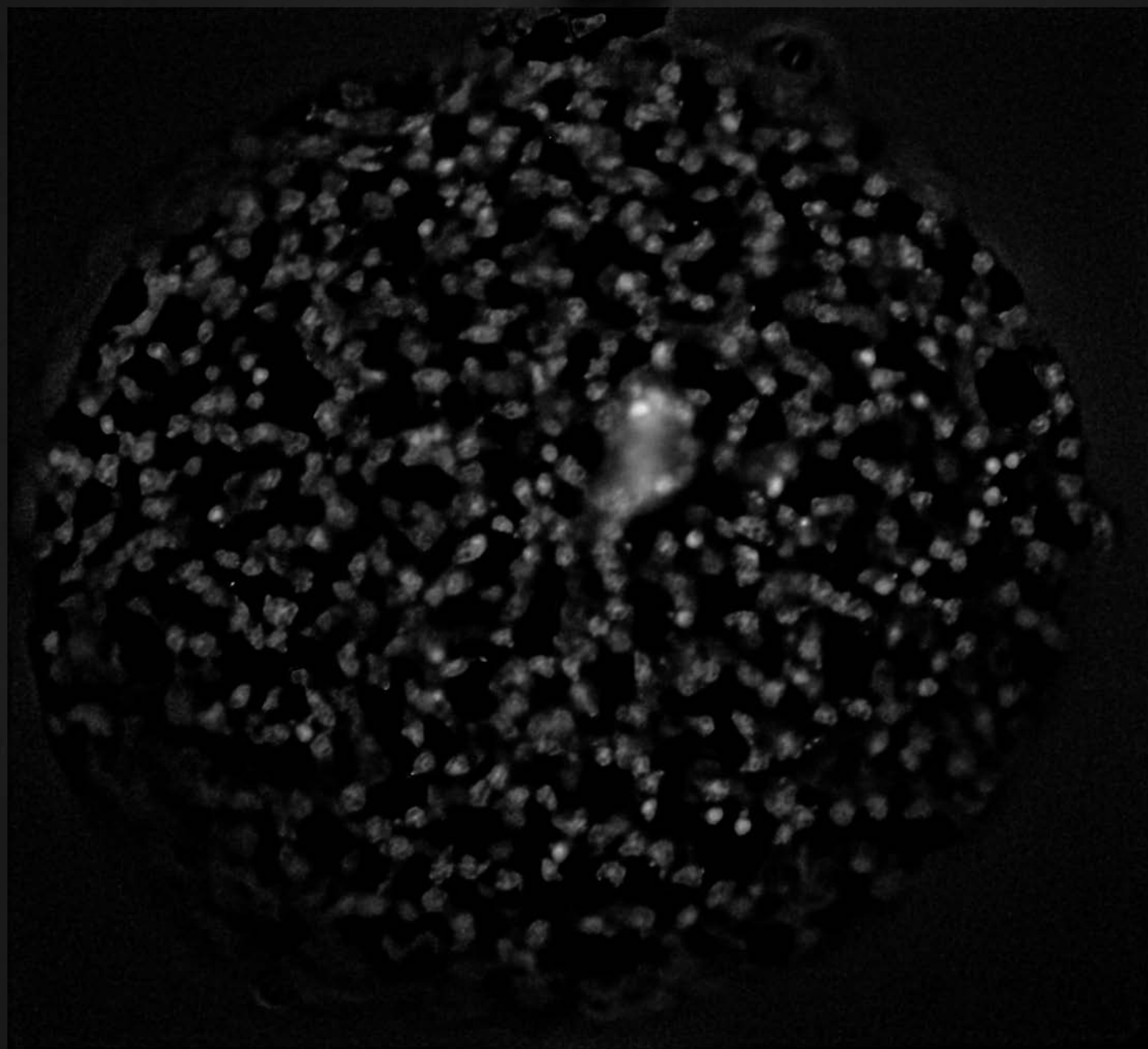

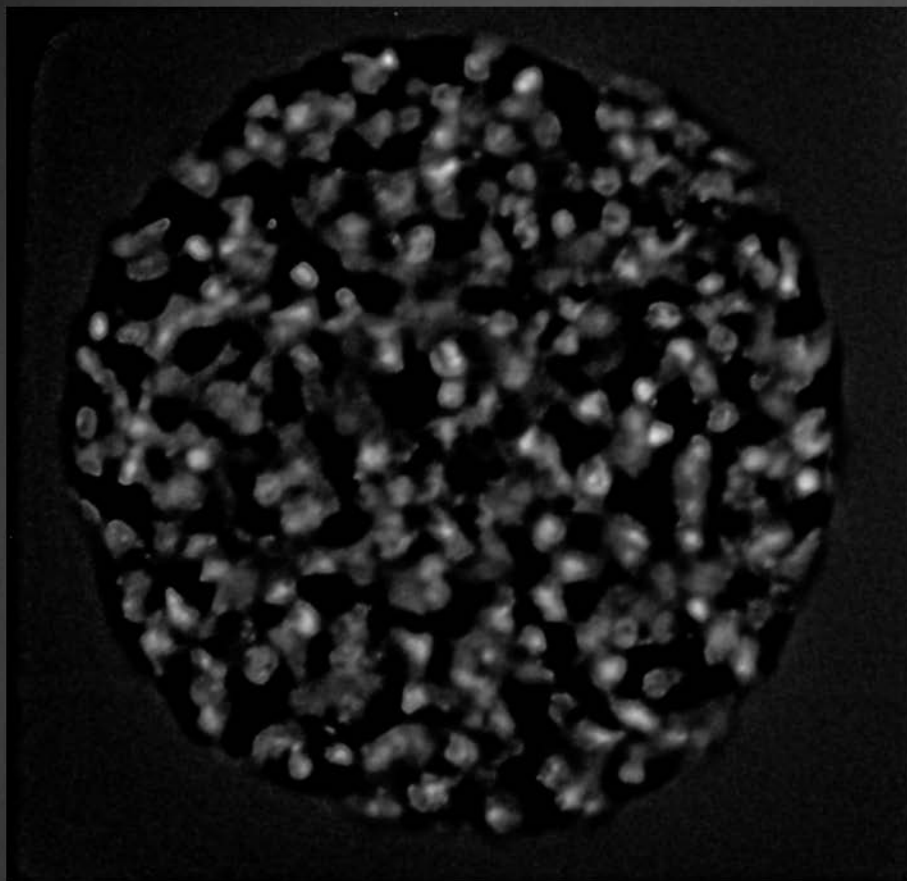

Supplement: S4 Raw images — (PDF) [file pone.0280746.s004.pdf]

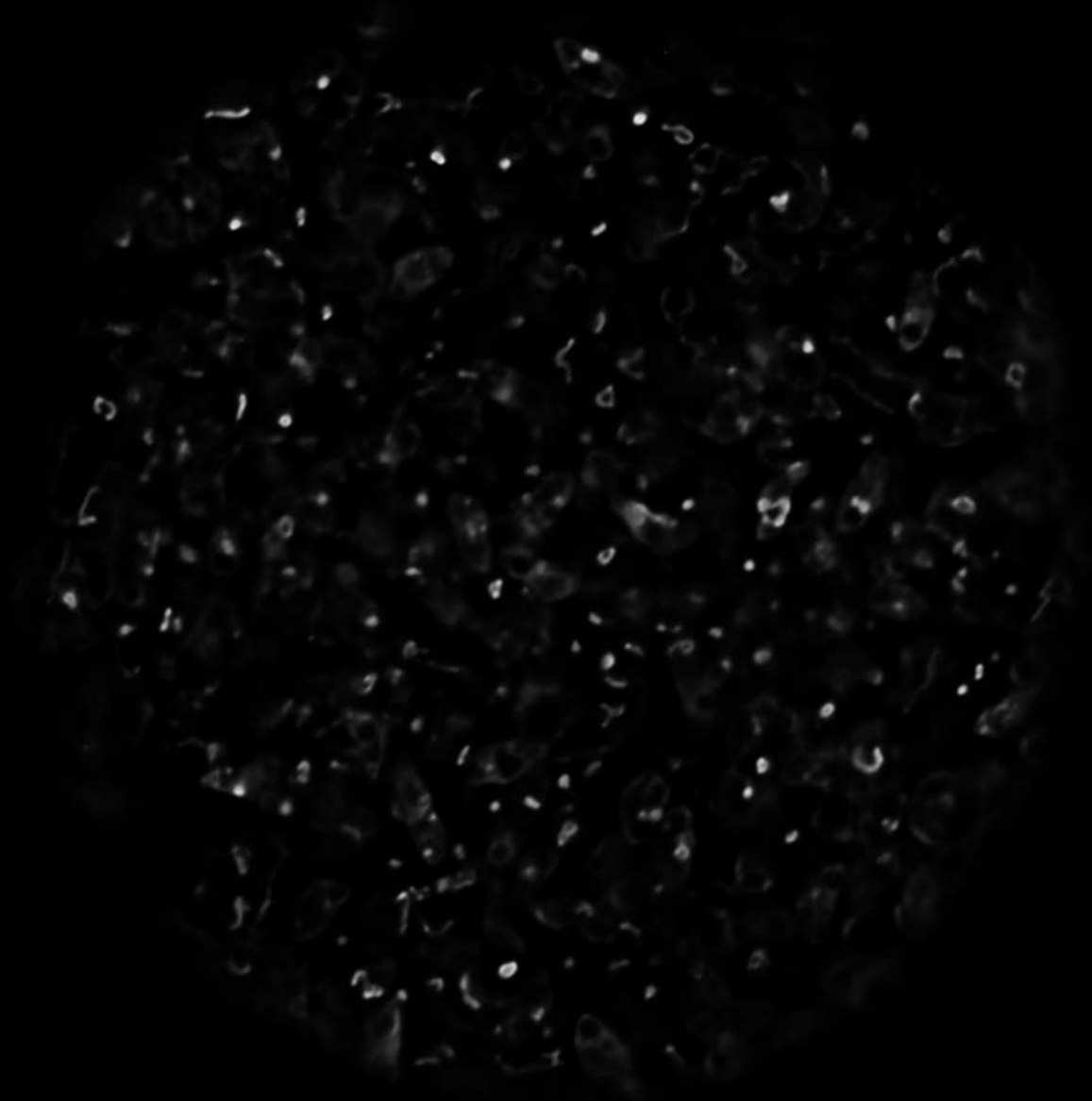

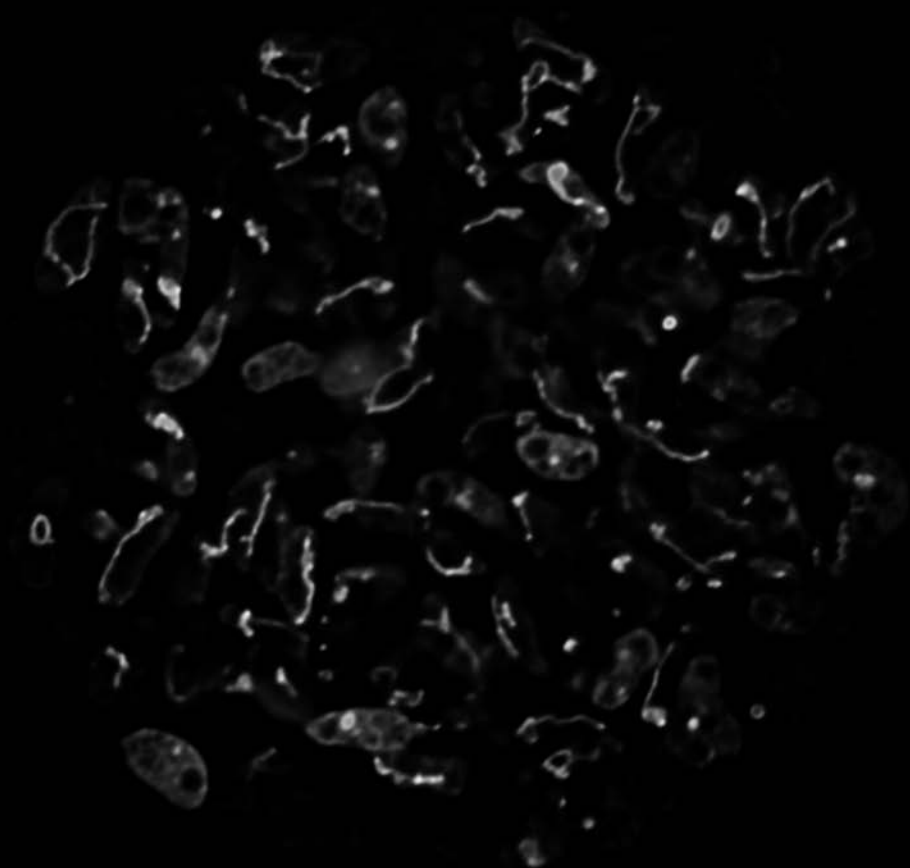

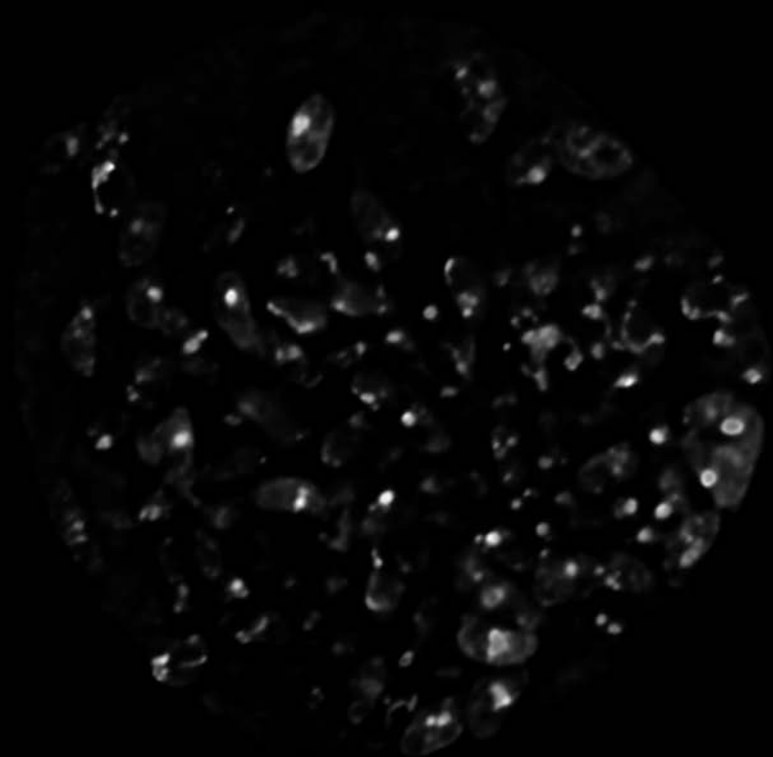

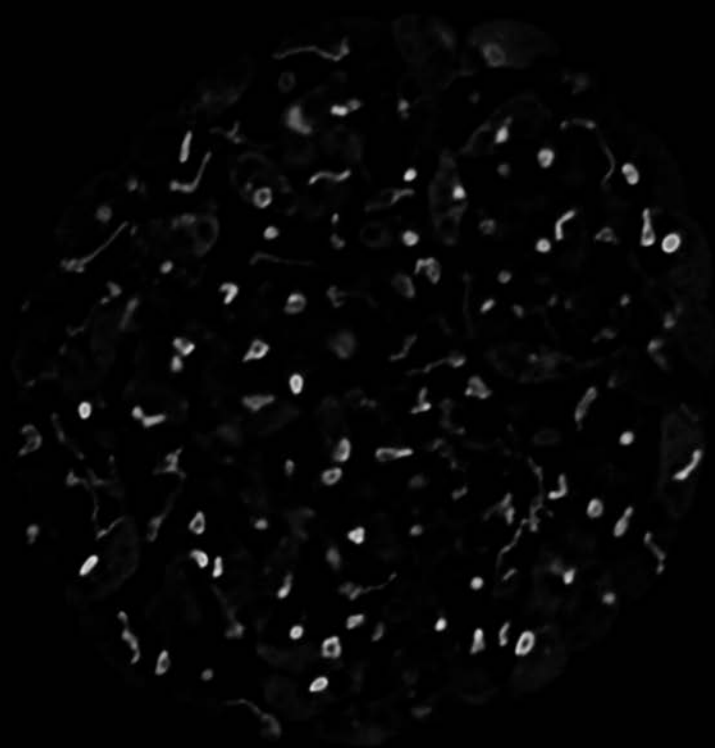

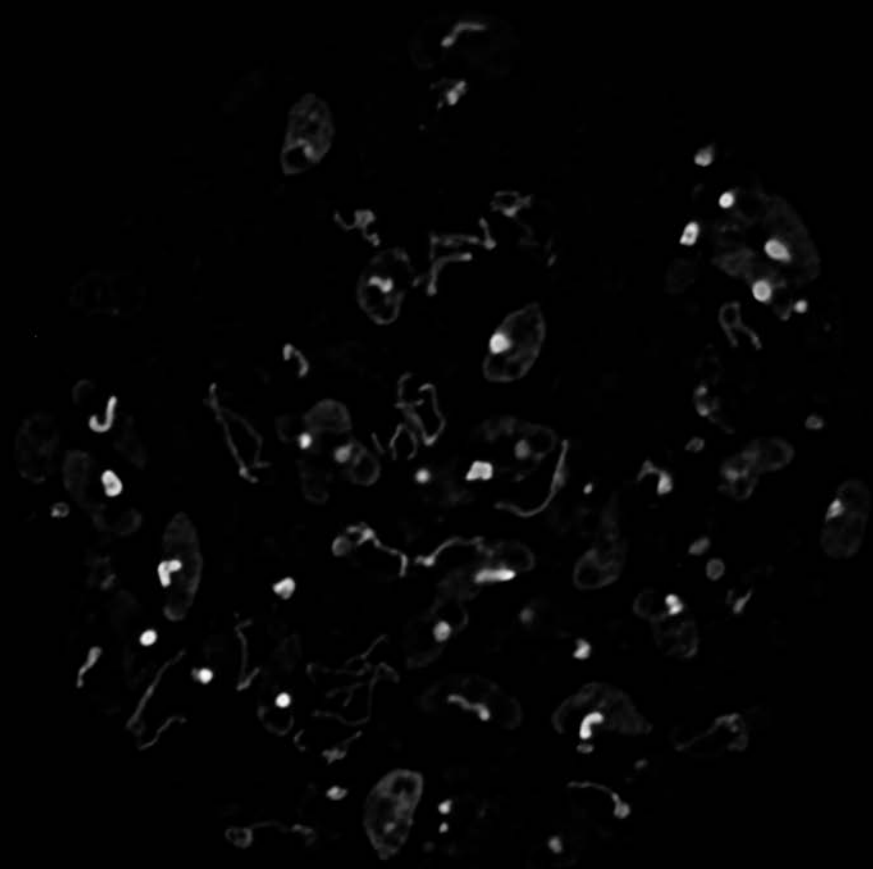

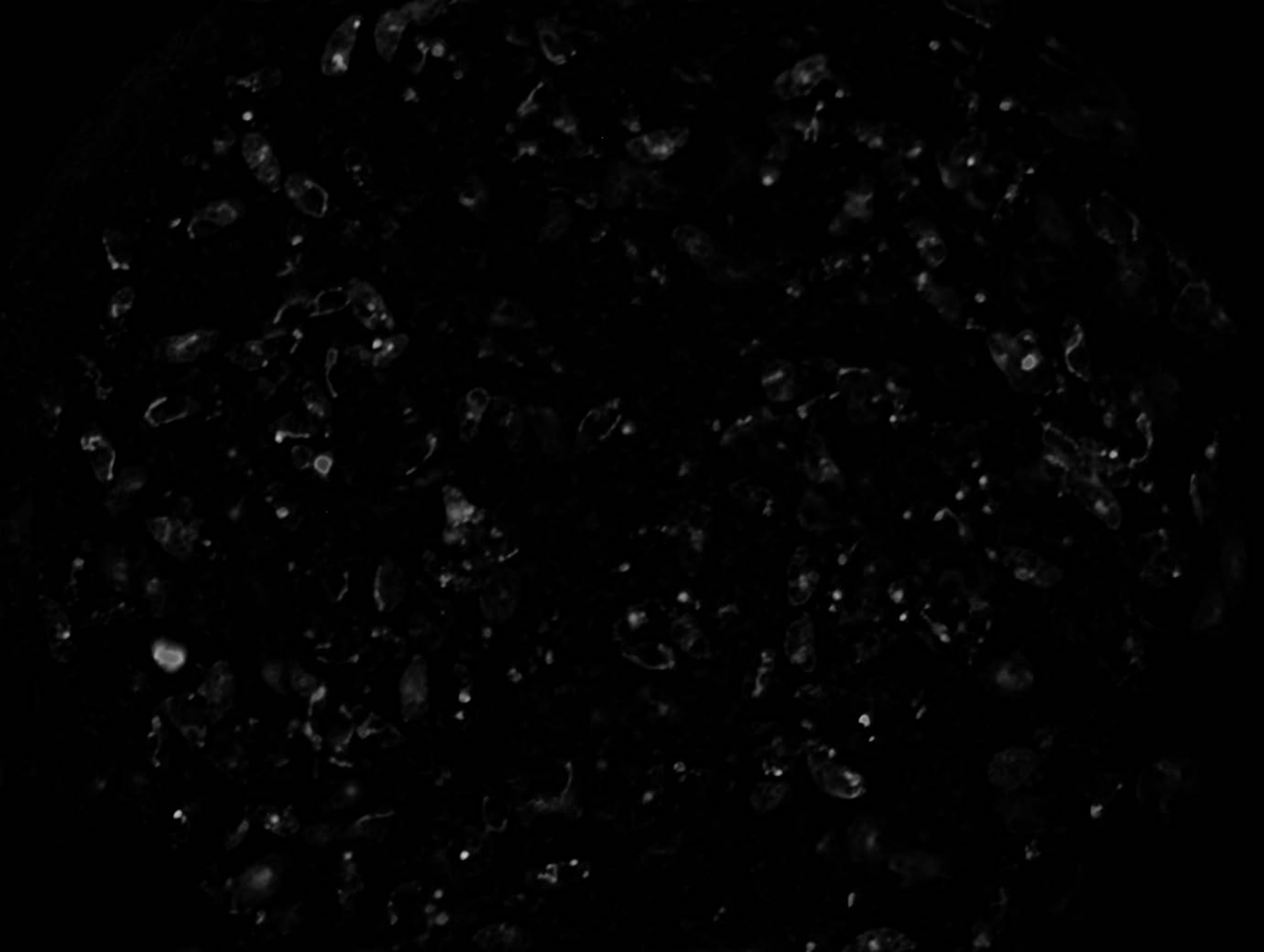

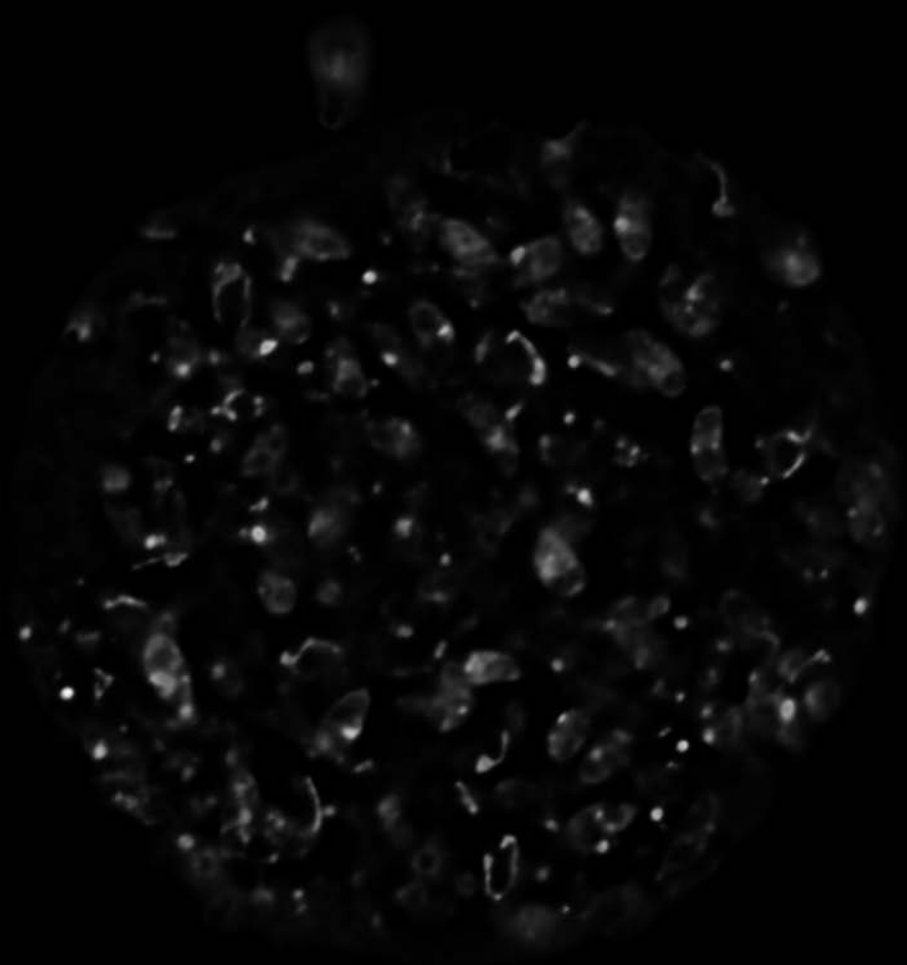

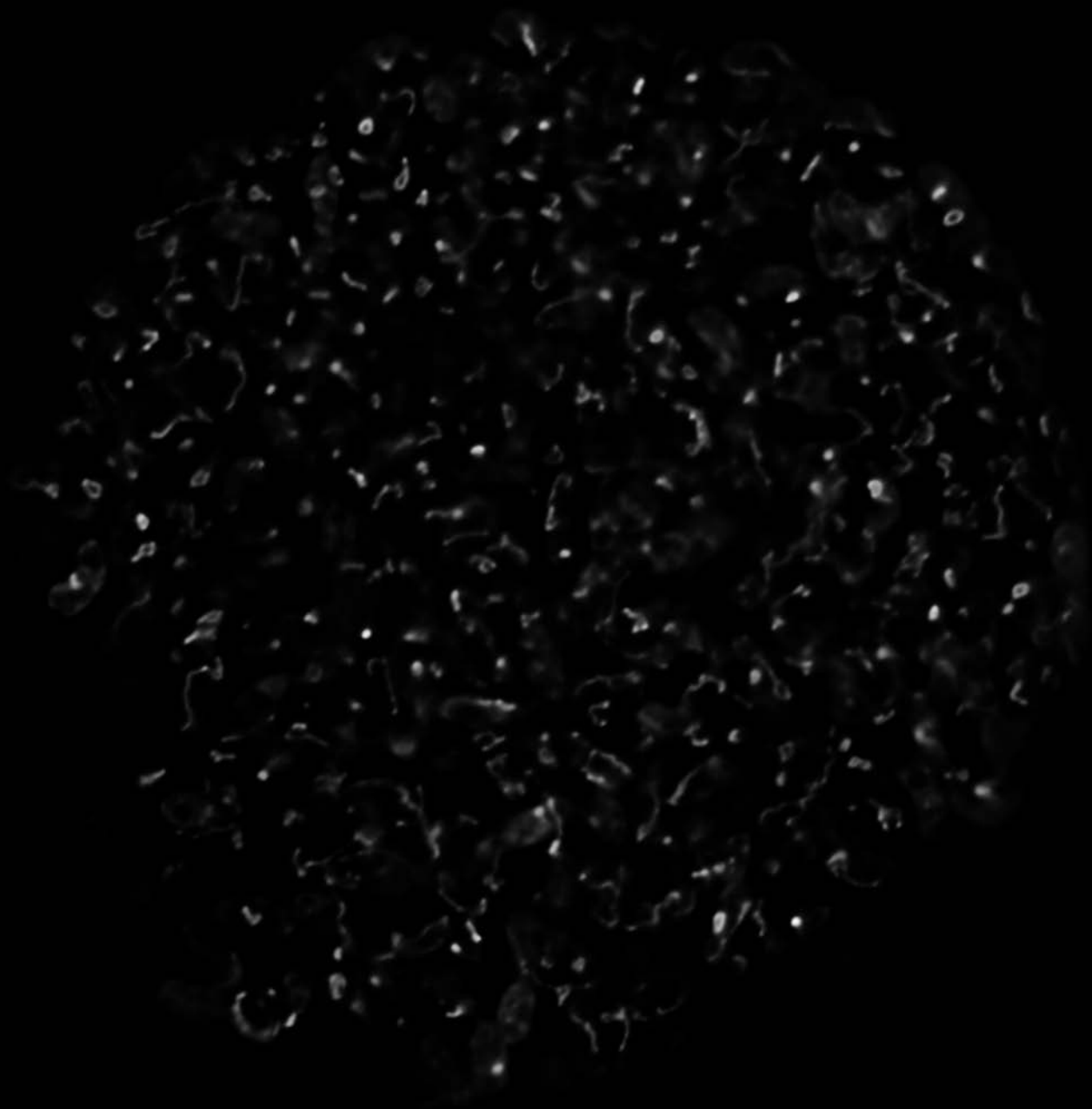

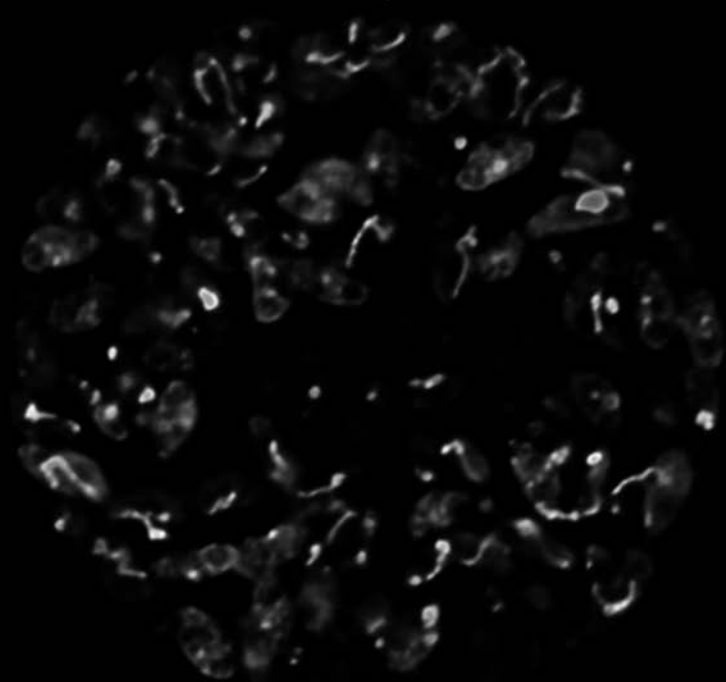

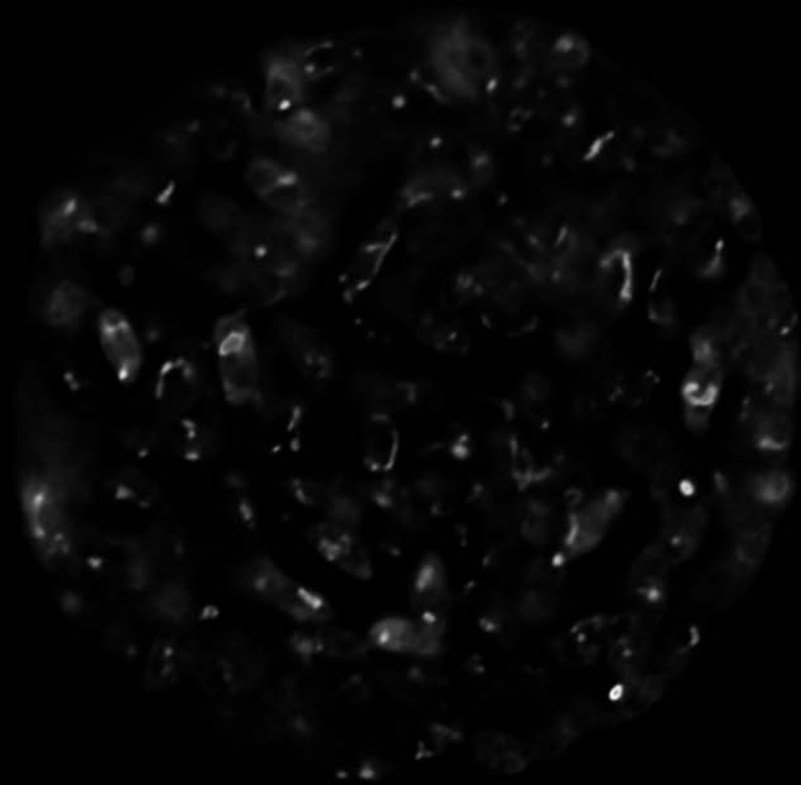

Supplement: S5 Raw images — (PDF) [file pone.0280746.s005.pdf]

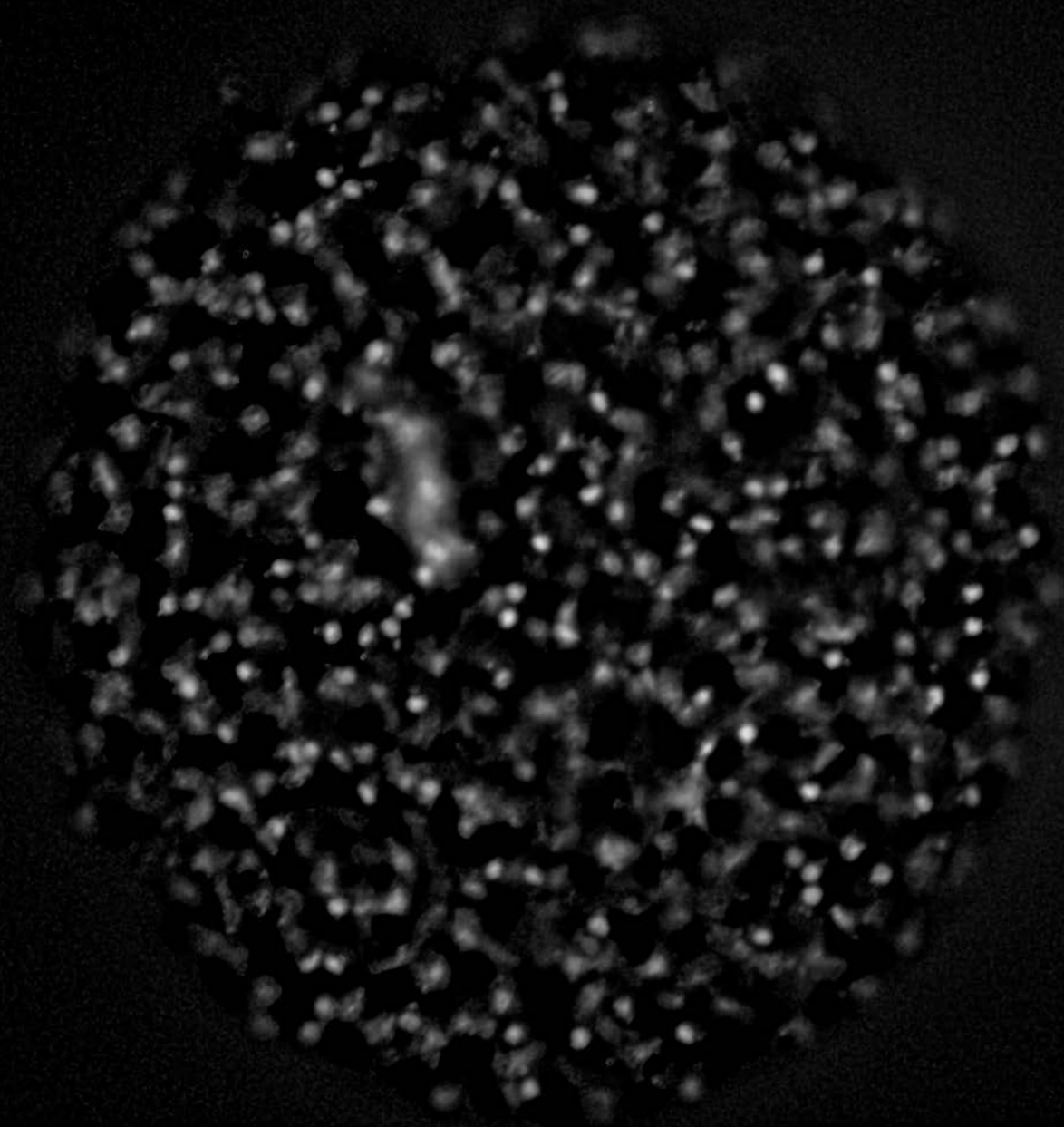

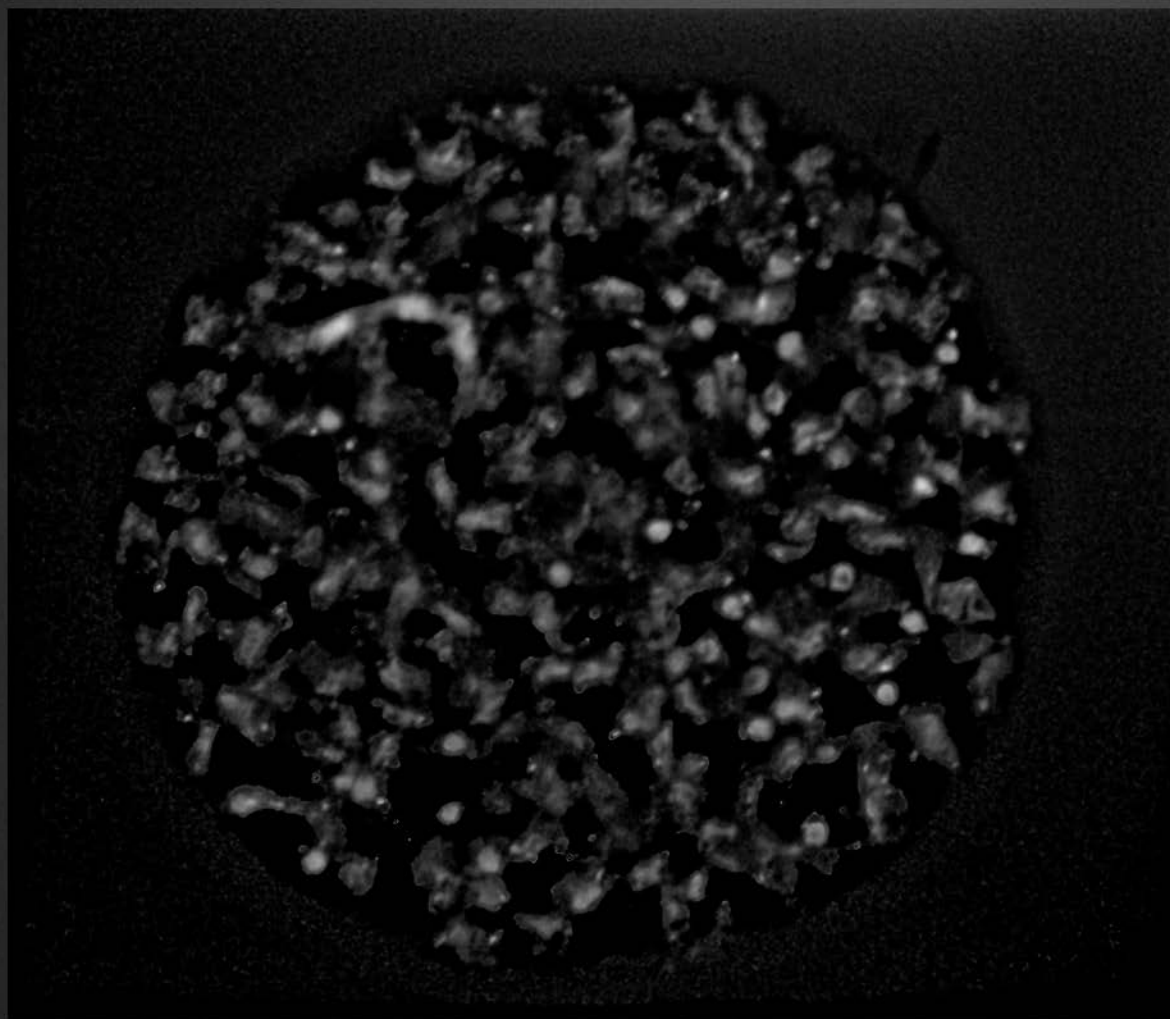

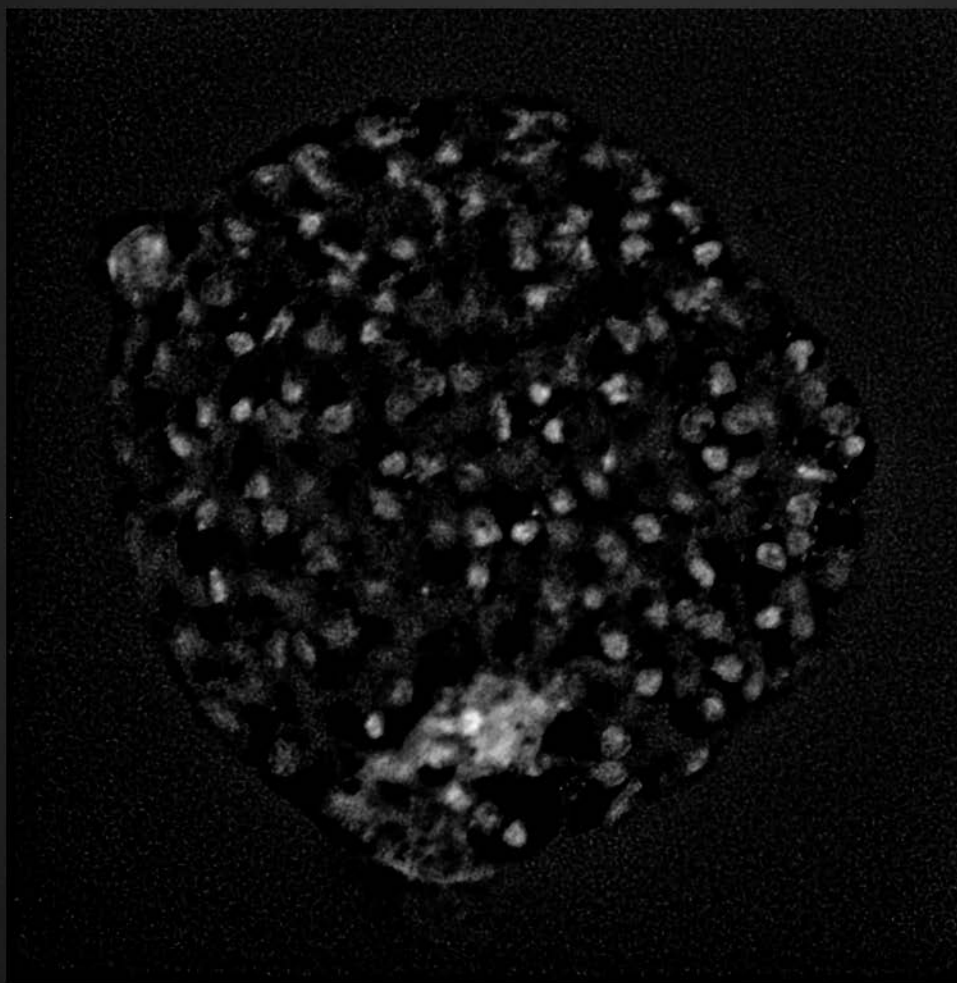

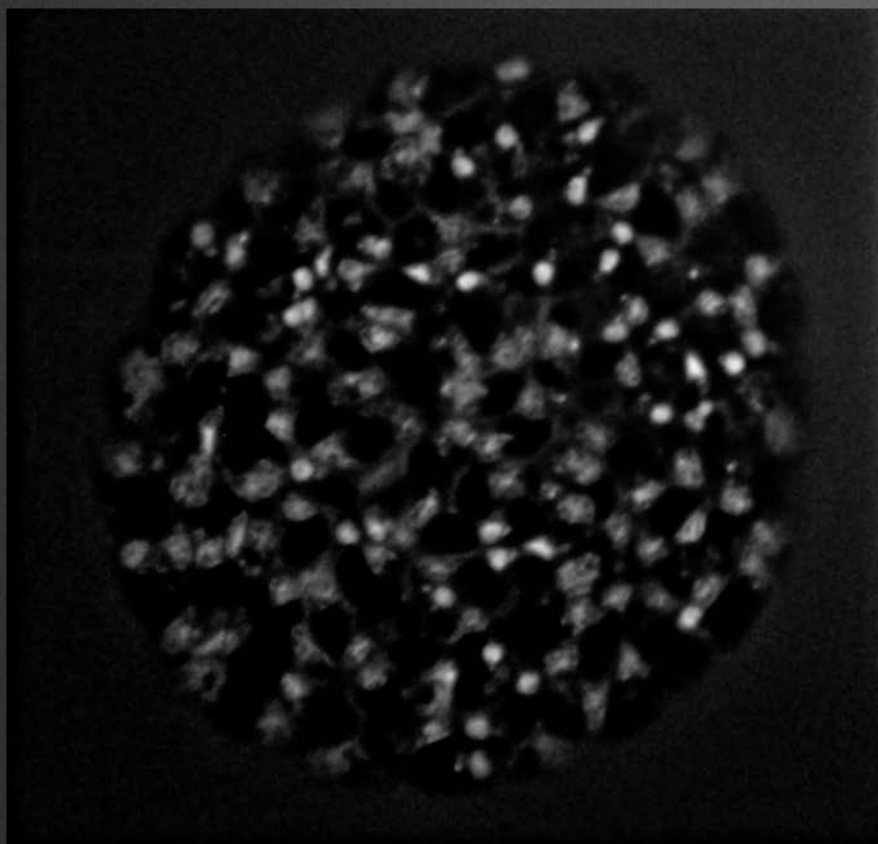

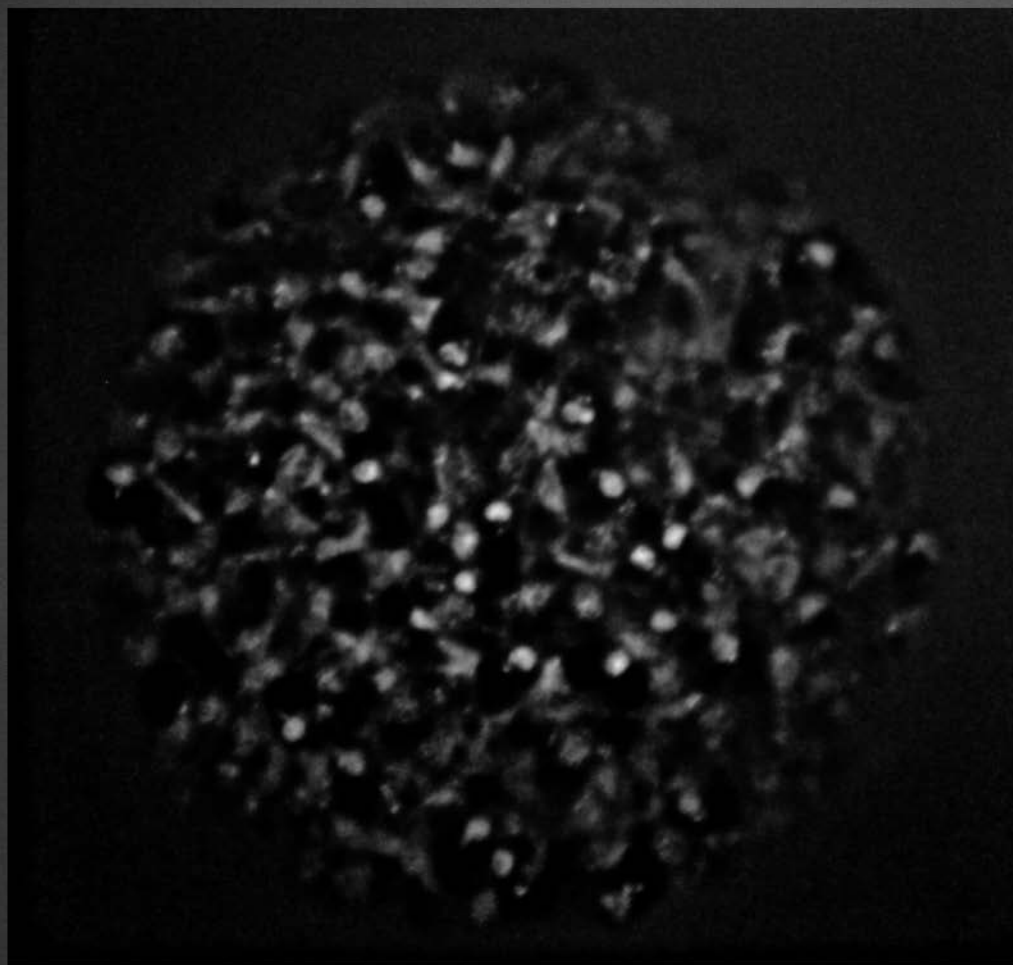

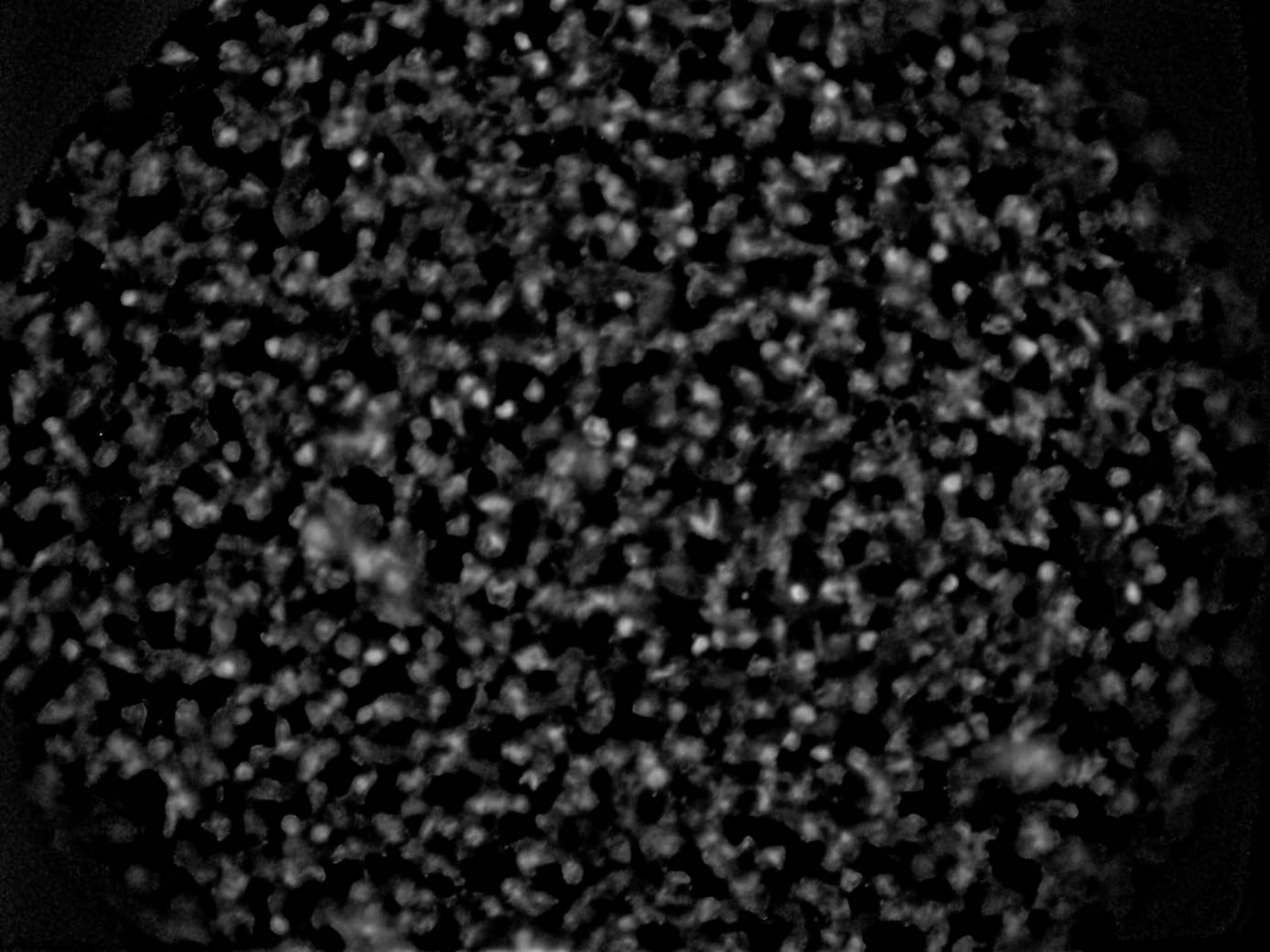

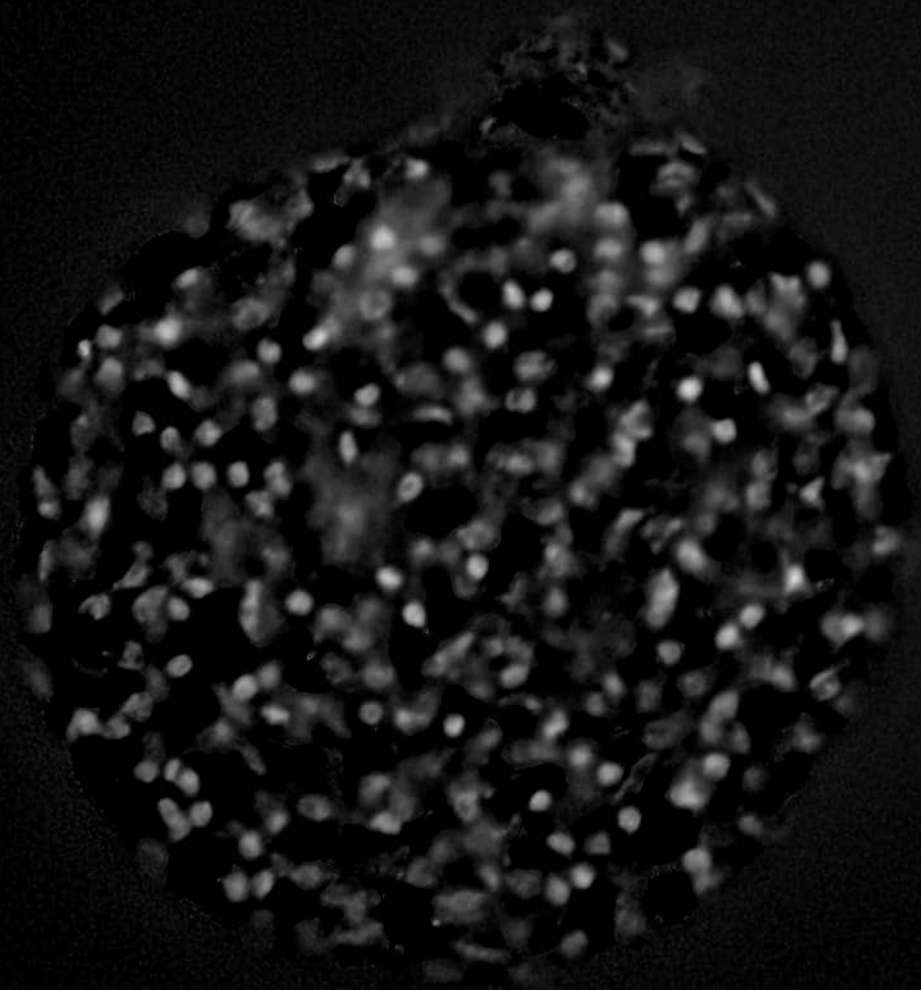

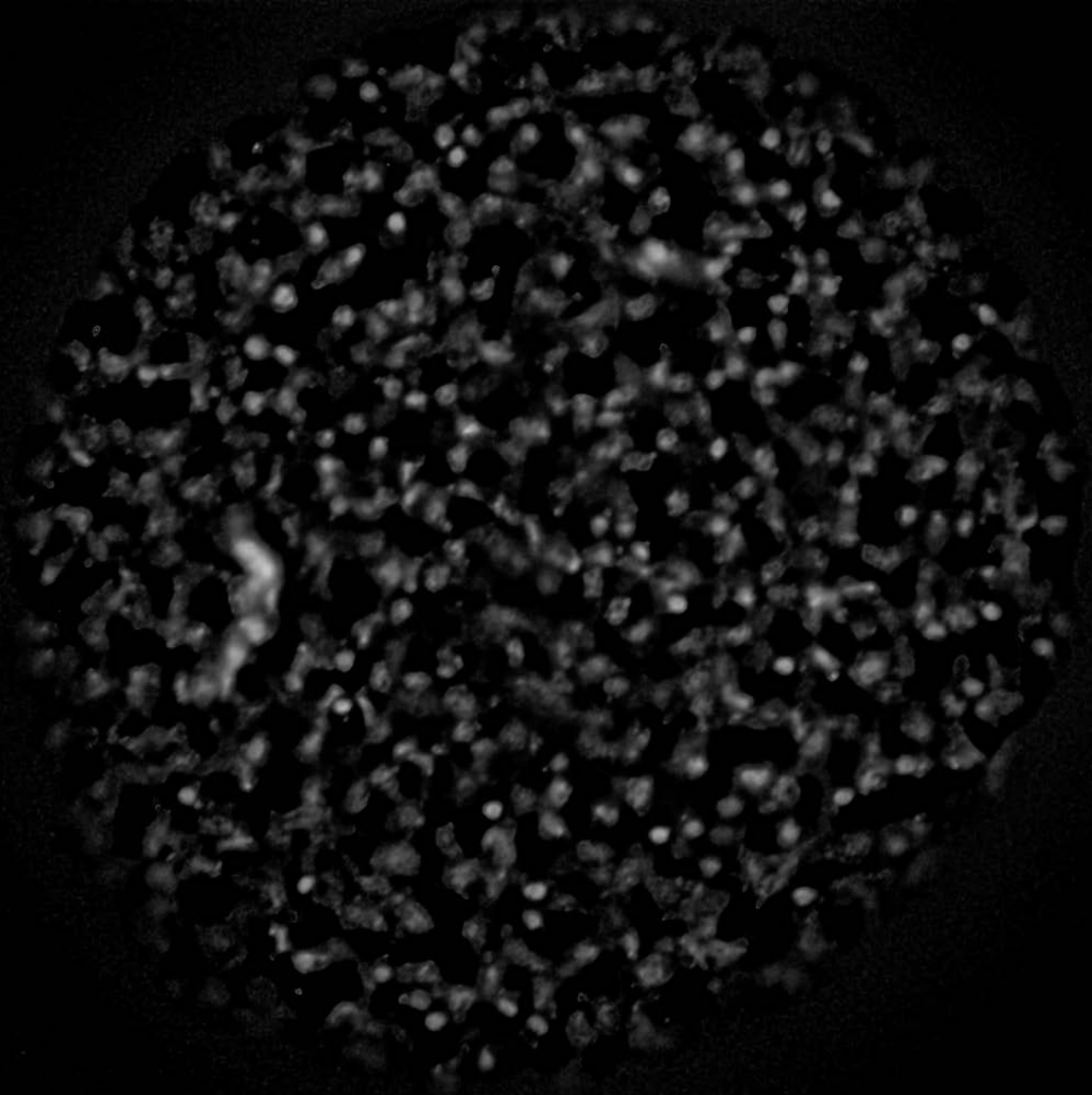

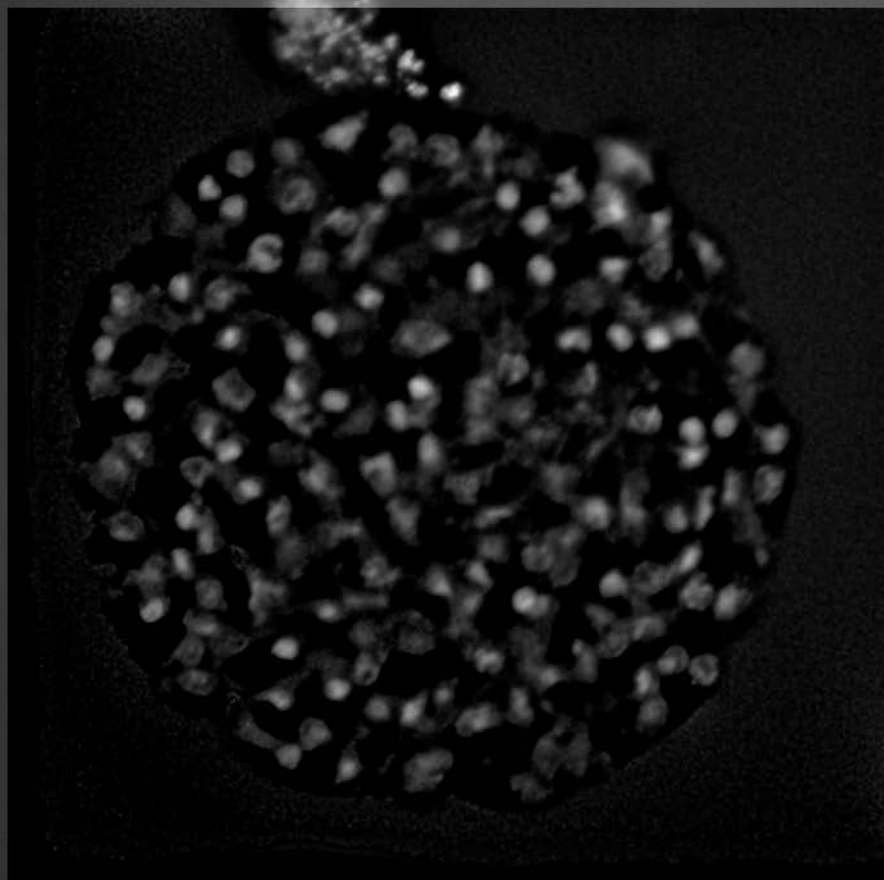

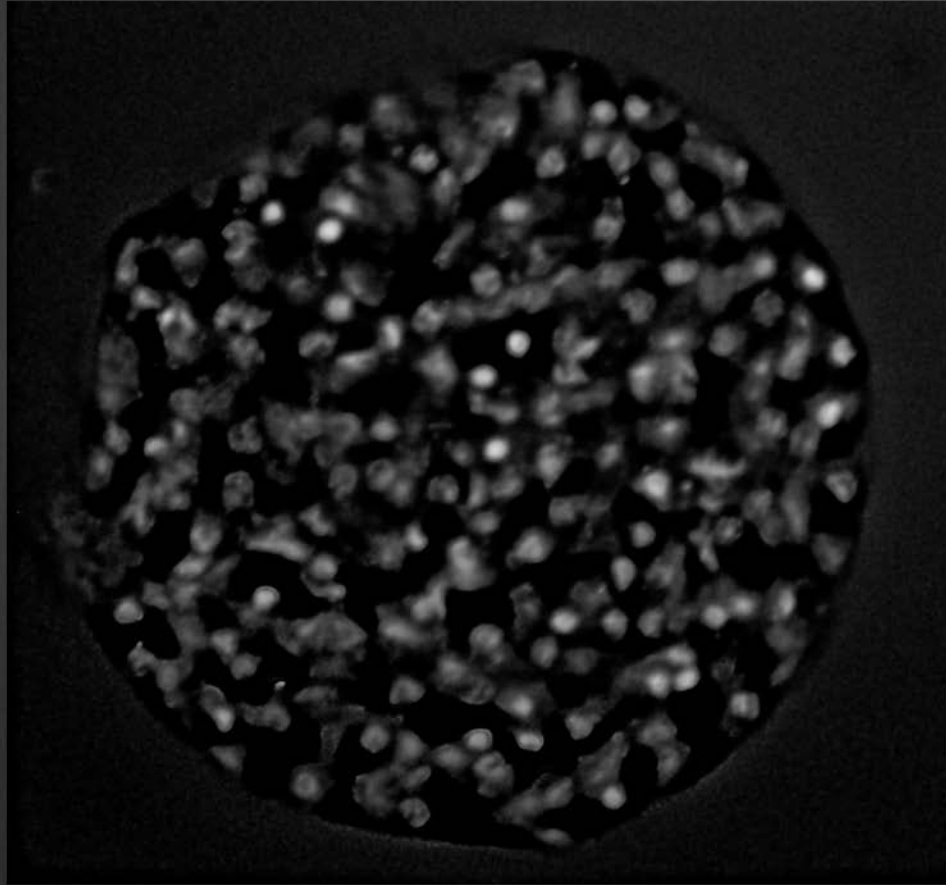

Supplement: S6 Raw images — (PDF) [file pone.0280746.s006.pdf]
